# Simulation and Data-Driven Modeling of the Transport Properties of the Mie Fluid

**DOI:** 10.1021/acs.jpcb.3c06813

**Published:** 2024-01-05

**Authors:** Gustavo Chaparro, Erich A. Müller

**Affiliations:** Department of Chemical Engineering, Sargent Centre for Process Systems Engineering, Imperial College London, South Kensington Campus, London SW7 2AZ, U.K.

## Abstract

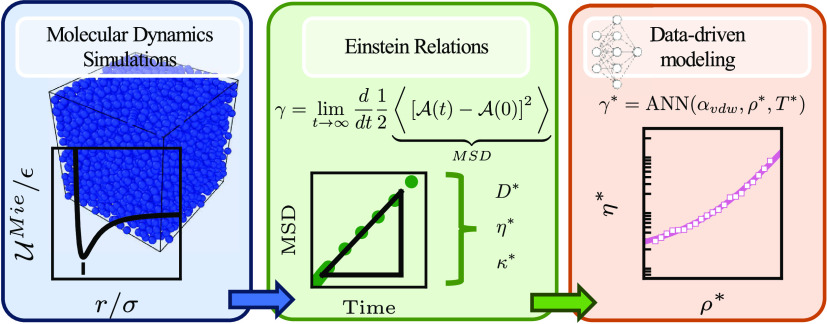

This work reports the computation and modeling of the self-diffusivity (D*), shear viscosity (η*), and thermal conductivity (κ*) of the Mie fluid. The transport properties were computed using equilibrium molecular dynamics simulations for the Mie fluid with repulsive exponents (λ_r_) ranging from 7 to 34 and at a fixed attractive exponent (λ_a_) of 6 over the whole fluid density (ρ*) range and over a wide temperature (T*) range. The computed database consists of 17,212, 14,288, and 13,099 data points for self-diffusivity, shear viscosity, and thermal conductivity, respectively. The database is successfully validated against published simulation data. The above-mentioned transport properties are correlated using artificial neural networks (ANNs). Two modeling approaches were tested: a semiempirical formulation based on entropy scaling and an empirical formulation based on density and temperature as input variables. For the former, it was found that a unique formulation based on entropy scaling does not yield satisfactory results over the entire density range due to a divergent and incorrect scaling of the transport properties at low densities. For the latter empirical modeling approach, it was found that regularizing the data, e.g., modeling ρ*D* instead of D*, ln η* instead of η*, and ln κ* instead of κ*, as well as using the inverse of the temperature as an input feature, helps to ease the interpolation efforts of the artificial neural networks. The trained ANNs can model seen and unseen data over a wide range of density and temperature. Ultimately, the ANNs can be used alongside equations of state to regress effective force field parameters from volumetric and transport data.

## 1 Introduction

The Mie potential is a simple, semiempirical interaction potential that considers both repulsive and attractive interactions. This potential has gained attention because of its flexibility in modeling thermophysical properties of a diverse range of fluids and fluid mixtures.^1^ For example, the Mie potential has been used as a model to represent the fluid phase equilibria of simple^2^ and asymmetric^3^ mixtures, biofuel blends,^4,5^ active pharmaceutical ingredients (API),^6,7^ cryogens with quantum effects,^8,9^ and polymers.^10,11^ Additionally, the Mie potential has been used to model the self-assembly of liquid crystals,^12^ superspreading of surfactants,^13^ asphaltene aggregation,^14^ and interfacial tensions.^15,16^ Furthermore, it has been used as a model to describe the transport properties, such as bulk^17^ and shear viscosities^18,19^ and diffusion coefficients^20^ of complex fluids. Formally, systems interacting through the Mie potential are governed by the following expression for the interaction energy:
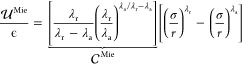
1Here,  is the interaction energy between two particles, ϵ is the interaction energy well depth, σ is the length scale, loosely associated with the effective particle diameter, and *r* is the center-to-center distance between two Mie particles. Finally, λ_r_ and λ_a_ are the repulsive and attractive exponents, respectively. It has been proven that for the description of fluid phase equilibria, the exponents of the Mie potential are conformal,^21^ implying that multiple combinations of the repulsive and attractive exponents can lead to the same macroscopic thermophysical properties. For simplicity, then, the attractive exponent is commonly taken as 6, in agreement with the London theory for dispersion forces,^22^ resulting in what is sometimes referred to as the (λ_r_, 6) Mie potential.^1^ Significantly, if the repulsive exponent is also set to 12, the Mie potential reduces to the well-known Lennard-Jones potential.

For design and engineering purposes,^23,24^ it is necessary to obtain accurate data for both thermodynamic properties (e.g., vapor pressure, phase equilibria, heat capacities) and transport properties (e.g., diffusivity, shear viscosity, thermal conductivity) of a fluid (or pseudo fluid in this case) over a wide range of conditions. The former is appropriately obtained from molecular-based equations of state (EoS) and empirical correlations. For the particular case of the Mie fluid, several EoSs that represent the volumetric properties are available. Among them are the SAFT-VR-Mie EoS^25^ and its group contribution version, the SAFT-γ-Mie EoS,^26^ which are based on perturbation theory. Reimer et al.^27^ proposed an EoS for the Mie fluid based on the UV-theory.^28^ An empirical EoS proposed by Pohl et al.^29^ can also model Mie fluids accurately. Recently, we have also proposed the FE-ANN EoS,^30^ which is an equation of state for the Mie fluid formulated as a physics-informed artificial neural network (ANN). In the same spirit as this contribution, the FE-ANN EoS was trained directly on molecular dynamics (MD) data and can very accurately predict the thermodynamic properties of the Mie fluid over a wide range of density and temperatures.

In contrast to the success in modeling volumetric (thermodynamic) properties, the modeling of the transport properties of the Mie fluid is scarcely covered in open literature. One of the challenges is that there is no commonly accepted framework for describing transport properties. Transport properties have been historically modeled using a combination of empirical, semiempirical, and theoretical-based approaches. Correlations based on a fluid’s density and temperature have been widely used by the National Institute of Standards and Technology^31^ to model transport properties of real fluids (see refs (32−34) to name a few) and have been adapted to model transport properties of molecular fluids, such as the truncated and shifted Lennard-Jones fluid.^35^ Empirical approaches can model the transport properties accurately, but in most cases, the approach lacks generality, and extrapolation should be avoided.

The transport properties have also been modeled with semiempirical approaches. For example, the shear viscosity of pure fluids and fluid mixtures has been related to the available free volume.^36^ Friction theory^37^ relates the shear viscosity to the balance between attractive and repulsive pressures. These two theories rely on using an EoS to either obtain the fluid density or the attractive/repulsive pressures. Following a similar pathway, entropy scaling^38^ has been postulated as a framework to link thermodynamic and transport properties. In this framework, a reduced transport property is considered a univariate function of the residual entropy, which is obtained directly from an EoS. Even though the concept of entropy scaling has its roots in an empirical observation, it can be derived from isomorph theory.^39,40^ One of the limiting issues with entropy scaling is that the theory is usually valid only for dense (liquid-like) phases, and there is a diverging behavior in the low-density regime. Furthermore, the functional relationship of the transport property with respect to the reduced entropy is, in most cases, obtained empirically.^41−44^ Thermodynamic (density) scaling, on the other hand, states that the transport property of a dense state is invariant with respect to the ratio of a scaled density and the temperature. Thermodynamic scaling also has its roots in isomorph theory.^39^ Ultimately, transport properties have also been modeled using theoretically based approaches. The options are scarce and mainly limited to the low-density region. The Chapman–Enskog theory,^45^ which can be used to obtain the transport properties of low-density fluids, has been extended to predict transport properties of the Mie potential.^20^ This last contribution shows that transport properties can be accurately predicted for real fluids using molecular parameters fitted only to equilibrium properties. Even with the promising results of the revised Enskog theory for Mie fluids,^20^ to the best of our knowledge, these predictions have yet to be verified by molecular simulation results.

Regardless of which modeling approach is to be used, it is necessary to rely on reference data for the transport properties of the Mie fluid. In the literature, transport property data over a wide range of density and temperatures have been collected and generated by Lautenschlaeger and Hasse^35^ for the Lennard-Jones fluid. Ŝlepaviĉius et al.^46^ generated self-diffusivity and shear viscosity data for a few Mie fluids. This generated database consists of about 1000 data points for the self-diffusivity and 270 data points for the shear viscosity for low to moderate densities and low to moderate supercritical temperatures. We still see the necessity of having a comprehensive and self-consisted database for transport properties, which also includes thermal conductivity and a wide range of densities and temperatures.

An ancillary objective of this work consists of adequately modeling the transport properties of the Mie fluid. Even though we value the recently revised Enskog theory approach,^20^ we recognize the limitations of using the theory at more dense conditions. For this reason, we have decided to test two approaches. The first empirical approach directly models a transport property based on its state conditions (density and temperature) using a machine learning approach. This data-driven approach has already been used to correlate the transport properties of pseudo and real fluids.^46−49^ A second semiempirical approach uses entropy scaling to model the transport properties. As the functionality between the input variables and the transport property is not known beforehand, we agree with Ŝlepaviĉius et al.^46^ that this is an ideal case for using machine learning to discover the structure–property relationships between the transport property and the input model.

The rest of this article is structured as follows. Section 2 consists of two parts. The first part details the transport property data generation from molecular dynamics simulations. The second part briefly describes the transport properties modeling approaches used in this work. In Section 3, the main results are shown and explained, including the assessment of the database and its data-driven modeling. Finally, in Section 4, the findings of this work are summarized and discussed.

## 2 Methodology

### 2.1 Molecular Dynamics Simulations

#### 2.1.1 Calculation of Transport Properties

In molecular dynamics (MD) simulations, the transport properties can be obtained either from nonequilibrium (NEMD) or equilibrium (EMD) simulations. Both approaches have advantages and disadvantages. In NEMD, the simulation is perturbed in a way that the transport property can be related to its fundamental definition, e.g., thermal conductivity from Fourier’s law^50^ and shear viscosity from the Newtonian fluid definition.^51^ In this approach, the transport property is obtained in a transient state. For this reason, the results are usually obtained with shorter simulation times. However, as an otherwise equilibrated simulation box is uniquely perturbed, either by a temperature or a momentum gradient, only one transport property can be computed at the time. On the other hand, in EMD, the simulation is integrated in time in its canonical state; hence, all of the transport properties can be obtained simultaneously, although at the expense of a longer simulation, as statistical uncertainty is considerable.

In EMD, two fundamentally equivalent methodologies exist to compute a transport property. In the Green–Kubo approach, eq 2a, a transport property (γ) is related to the integral of a time-correlation function of a dynamical variable . Similarly, the so-called Einstein method, eq 2b, relates the same transport property to the mean squared displacement (MSD) of the variable .
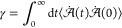
2a
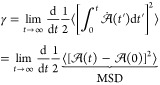
2bIn eq 2a and 2b, the ⟨···⟩ brackets refer to an ensemble average. Both approaches require sampling the property  (or ) for a sufficient time to obtain reliable statistics. The integrand in the Green–Kubo approach method slowly decays to zero regardless of the simulation time^52,53^ and, for that reason, time-decomposition methods have been proposed to improve the reliability of the results.^54^ On the other hand, the validity of the Einstein method can easily be checked by plotting the MSD as a function in a log–log plot. The Einstein formulation is only valid if the slope of the MSD curve equals to one.^55^ This latter approach is particularly convenient for simple, isotropic fluids and is used herein.

The self-diffusion coefficient (*D*) of a pure species is obtained as the averaged mean squared displacement of all of the particles.
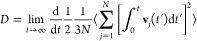
3a
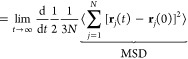
3bIn eqs 3a and 3b, *t* is the correlation time, *N* is the number of particles, and **r**_*j*_ and **v**_*j*_ refer to the position and velocity of the *j*th particle, respectively. The velocity integral in eq 3a corresponds to the particles’ position (eq 3b), which is readily available when integrating molecular dynamics simulations.

The shear viscosity is directly related to the time correlation of the pressure tensor. In the case of anisotropic fluids, the shear viscosity (η_*ij*_) on the direction *ij* is obtained as follows:
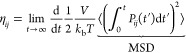
4Here, *V* corresponds to the total volume of the simulation box, *k*_b_ is the Boltzmann constant, *T* is the simulation temperature, and *P*_*ij*_ is the off-diagonal component of the pressure tensor in the direction *ij*. In the case of isotropic fluids, one can improve statistics by computing the shear viscosity (η) from the average of all of the components of the traceless pressure tensor (*P*_*ij*_^os^), as follows:^56^
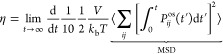
5where,
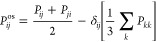
Here, δ_*ij*_ is the Kronecker delta function. The extra factor of 10 in the denominator of eq 5 results from the 3/3 contribution from each one of the six off-diagonal terms of the traceless pressure tensor, plus the 4/3 implicit contribution of each diagonal term (i.e., 6·3/3 + 3·4/3 = 10).

Finally, the isotropic thermal conductivity (κ) is related to the average heat flux, as shown below.
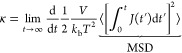
6In eq 6, *J* is the average heat flux. The heat flux vector (**J**) can be obtained for systems interacting through pairwise interaction potentials as follows:
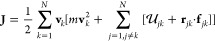
7Here, *N* corresponds to the total number of particles, **v**_*k*_ is the velocity vector of the particle *k*, and , **f**_*jk*_, and **r**_*jk*_ are the interaction energy, force, and distance vector between the particles *j* and *k*.

#### 2.1.2 Finite-Size Effects

Molecular simulations require periodic boundary conditions to mimic infinite-sized systems.^57,58^ However, it is well known that the calculation of transport properties may suffer from finite-size effects.^55,59−62^ For example, the computed diffusion coefficient using eqs 3a and 3b scales linearly with the inverse of the simulation box size.^59,60^ In the case of a cubic simulation box (i.e., *L* = *L*_*x*_ = *L*_*y*_ = *L*_*z*_), Yeh and Hummer^60^ derived the following expression to obtain the self-diffusivity of a virtually infinite size system (*D*_∞_).
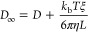
8Here, *D* is the self-diffusivity obtained from eqs 3a or 3b, ξ is a dimensionless universal constant equal to 2.837298, *L* is the simulation box length, and η is the shear viscosity computed from eq 5. We assume that neither the shear viscosity nor the thermal conductivity have appreciable finite-size effects.^55,60^

#### 2.1.3 Molecular Simulation Setup

Large-scale Atomic/Molecular Massively Parallel Simulator (LAMMPS) software^63^ is used to compute transport properties. The simulations are run using reduced units or equivalently to setting the Boltzmann constant (*k*_b_), potential well depth (ϵ), the shape parameter (σ), and Mie particle mass (*M*) to unity. Thermophysical properties reported in these dimensionless units are characterized by the superscript “*”. For completeness, the definition of selected thermophysical properties in reduced units is given in Table 1.

**Table 1:** Definition of Physical Quantities in Reduced Units

quantity	symbol	units	reduced quantity^a^^,^^b^^,^^c^
time	τ	s	
mass	*m*	kg mol^–1^	m* = *m*/*M*
density	ρ	mol m^–3^	ρ* = ρ*N*_a_σ^3^
temperature	*T*	K	T* = *Tk*_b_/ϵ
pressure	*P*	Pa	P* = *P*σ^3^/ϵ
self-diffusion	*D*	m^2^ s^–1^	
shear viscosity	η	Pa s	
thermal conductivity	κ	W m^–1^ K^–1^	

a Superscript (*) refers to a property in reduced units.

b *N*_a_ = 6.022142·10^23^ mol^–1^ is the Avogadro constant, *k*_b_ = 1.3806488·10^–23^ J K^–1^ is the Boltzmann constant, and *M* is the molar mass.

c σ is the shape parameter, and ϵ is the potential well depth of the Mie potential (see eq 1).

MD simulations are performed in a cubic simulation box with 4096 Mie particles. This relatively large number of particles is chosen to minimize any possible finite-size effects of the shear viscosity and thermal conductivity, for which no analytical correction is available. The MD simulations are run using a cutoff radius of *r*_c_ = 5σ, and no long-range corrections are applied. The simulations are run with a time step of Δτ* = 0.002 τ*. The simulation is first equilibrated for 10^6^ timesteps in the canonical (NVT) ensemble using the Nosé–Hoover thermostat^64^ with a time constant of 100 Δτ*.

Once the system is equilibrated at a desired temperature, the simulation is integrated in the microcanonical (NVE) ensemble for 2.9·10^6^ timesteps using the velocity Verlet algorithm.^65^ During this production stage, statistics of the systems are accumulated every 1000 timesteps. The MSDs to compute self-diffusivity, shear viscosity, and thermal conductivity are obtained using the OCTP plugin.^55^ As recommended by Jamali et al.,^55^ the self-diffusivity is sampled every 1000 timesteps, and the viscosity and thermal conductivity are sampled every 5 timesteps. For each simulation, the MSDs are obtained for ten different starting points separated by 10^5^ timesteps between each other.

#### 2.1.4 Database Curation

The transport properties are obtained as follows using standard NumPy(66) and SciPy(67) functions. First, the logarithm of time and the logarithm MSD are fitted to a cubic spline. The derivative of the fitted spline is computed, and the region where the slope is 1 ± 0.075 is filtered. From this region, the corresponding transport property is computed using eqs 3b, 5, or 6 for the self-diffusivity, shear viscosity, or thermal conductivity, respectively. The procedure is repeated for each one of the ten computed MSDs, from which mean values and standard deviations are obtained for each state point.

The produced database is filtered in a two-step process. First, high variance data points in which the ratio between the standard deviation, σ_γ*_, and the transport property value, γ*, is greater than 0.2 (i.e., σ_γ*_/γ* > 0.2) are disregarded. Second, the LocalOutlierFactor(68) method implemented in Scikit-Learn(69) is used to disregard possible outliers. This outlier detection method provides scores that measure the “outlierness” of a data point. A regular data point is expected to have a score close to 1, while an outlier will have a “big” score. In the original publication, Breunig et al.^68^ suggested a value of 1.5 as a threshold to identify outliers. However, this value might be unsuitable for some data sets. For this reason, in this work, the threshold to filter outliers is obtained by fitting the total population of scores to a probability distribution. As most of the data points are normal, the mean of the distribution is close to 1. Then, the threshold is obtained from the upper bound of the 95% confidence interval of the distribution.^70^ An adequate distribution that fits the scores is found using the fitter(71) Python package.

### 2.2 Modeling of Transport Properties

In general, a transport property (γ*) of the Mie fluid needs to be identified by a Mie fluid’s descriptor and by its state conditions (i.e., density and temperature). While in general, the Mie fluid is characterized by both its repulsive (λ_r_) and attractive (λ_a_) exponents, without any loss of generality, it is more convenient to use the van der Waals constant (α_vdW_),^21^ defined as follows:
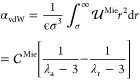
9In principle, a Mie fluid’s transport property could be considered a function of the van der Waals constant (α_vdW_), density (ρ*), and temperature (T*), i.e., γ* = γ*(α_vdW_, ρ*, T*). Alternatively, Rosenfeld^38^ suggested that a scaled transport property (γ̃*) is a function of the residual entropy (S^*,res^), i.e., γ̃* = γ̃*(α_vdW_, −S^*,res^). Within this framework, the scaled properties are given in eqs 10a–10c.
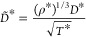
10a
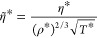
10b
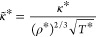
10cThe residual entropy of the Mie fluid is obtained here from an equation of state that models the residual Helmholtz free energy (A^*,res^) using artificial neural networks (FE-ANN EoS).^30^ The FE-ANN EoS is defined as follows:
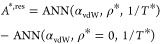
11The FE-ANN EoS evaluates the same artificial neural network twice, at the given density ρ*, and ρ* = 0, thus fulfilling the ideal gas law limit. The FE-ANN EoS satisfies the Maxwell relations and accurately models the Mie fluid’s first- and second-order derivatives over a vast range of densities and temperatures. The residual entropy is directly obtained by taking the isochoric temperature derivative of eq 11 as follows: S^*,res^ = (∂A^*,res^/∂T*)_ρ*_.

Regardless of which modeling approach has been used (γ*(α_vdW_, ρ*, T*) or γ̃*(α_vdW_, −S^*,res^)), the exact functionality to link the descriptors to the transport property is not known. For this reason, empirical combinations of exponential and polynomial expansions have been used in the literature.^35,41,44^ In this work, the transport property’s functionality is “learned” by using artificial neural networks (ANNs), that is,
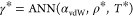
12a
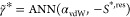
12b

#### 2.2.1 Artificial Neural Networks

Artificial neural networks (ANNs)^72^ are deep learning algorithms suitable for classification and regression problems.^73^ An ANN consists of a series of linear transformations followed by a nonlinear transformation (a.k.a. activation function). An *n*-layered ANN is mathematically represented as follows:
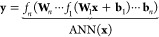
13In eq 13, **x** and **y** are the input and output features of the ANN, respectively. The *i*th layer in the ANN consists of a linear transformation done by the weight matrix **W**_*i*_ and the bias vector **b**_*i*_. The dimension of the weight matrix and bias vector are (*m*_*i*_ × *n*_*i*–1_) and *m*_*i*_, respectively, where *m*_*i*_ is the number of neurons on the *i*th layer and *n*_*i*–1_ is the dimension of the output vector from the previous layer. The linear transformation is followed by the activation function *f*_*i*_, which adds nonlinearity to the model.^73^ In this work, the hidden layers use the tanh activation function (*f*(*x*) = tanh(*x*)), while the output layer can use either the linear (*f*(*x*) = *x*) or softplus (*f*(*x*) = ln(1 + exp(*x*)) activation functions. This selection of activation functions ensures the output of the ANN is continuous and differentiable with respect to the inputs.

The training of the ANN consists of updating the weights (**W**_*i*_) and biases (**b**_*i*_) of each layer to minimize the following loss function ().
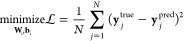
14In eq 14, **y**_*j*_^true^ and **y**_*j*_^pred^ are the *j*th samples of the true and predicted output features, respectively. The minimization is done by using the Adam method.^74^ This method consists of a modified gradient descent step that carries information from previous iterations (a.k.a. momentum). In a gradient descent step, the ANN’s parameters are updated as follows:
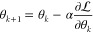
15Here, θ_*k*_ is an ANN’s parameter (weight or bias) at the step, *k* and α is the learning rate. The derivative of the loss function with respect to the parameter () is obtained using automatic differentiation.^75^ The ANN’s training can be intensive in memory use; for this reason, the minimization is performed in mini-batches of data. An epoch is completed when the minimization has gone through the entire training data set.

The data set is split into a training and a validation data set using a 90/10 split ratio. The ANNs are trained using the training data set, while the validation data set is used to assess if the ANN can generalize to unseen data.

The architecture of ANN, i.e., the number of layers, the number of neurons per layer and the activation function, the minimization algorithm, the learning parameter, and the number of epochs and size of the mini-batches, are hyperparameters of the ANN. In this work, the hyperparameters of the ANNs are optimized. However, the following standard practices are imposed to reduce the dimensionality of the hyperparameter optimization: the hidden layers use the tanh activation function and are restricted to have the same number of neurons, the output layer can use either the linear or softplus activation function, the Adam optimizer is used for training, the batch size is set to 32, and the number of epoch is set to 1000.

In this work, the ANNs are implemented using flax,^76^ and the ANNs are trained using optax, which is part of the DeepMind JAX Ecosystem.^77^ The hyperparameter tuning is performed using the Tree-structured Parzen Estimator^78^ included in Optuna.^79^

## 3 Results and Discussion

### 3.1 Transport Properties Database

**Figure 1:**
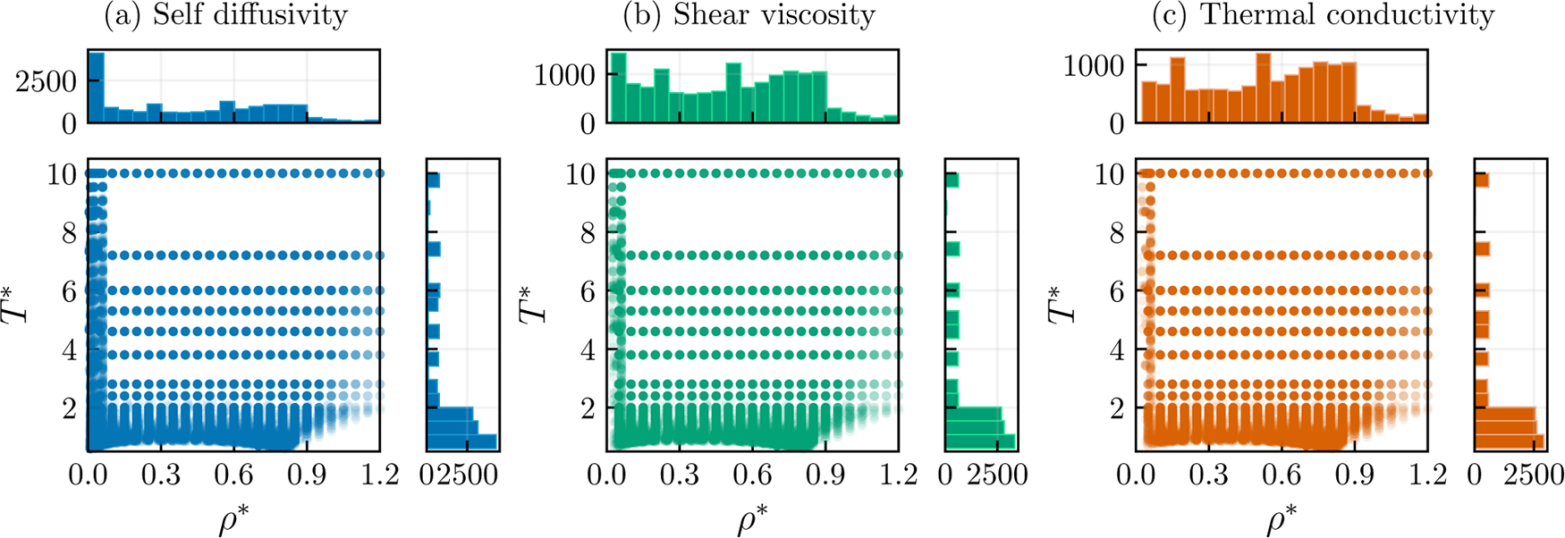
Distribution (ρ*–T*) of the Mie fluid transport properties database. (a) Self-diffusivity, (b) shear viscosity, and (c) thermal conductivity.

Transport properties for 28 Mie fluids were obtained for densities (ρ*) from 5 × 10^–3^ up to 1.2 and temperatures (T*) from 0.6 up to 10.0. The attractive exponent (λ_a_) was set to 6, whereas the repulsive exponent (λ_r_) varied for integers from 7 up to 34. This combination of exponents corresponds to α_vdW_ parameters (eq 9) in the range from 0.53 to 1.47, which covers most relevant small molecular weight fluids.^21^ After disregarding high variance results and possible outliers, as mentioned in Section 2.1, the final database consists of 17,212, 14,288, and 13,099 data points for the self-diffusivity, shear viscosity, and thermal conductivity, respectively. This data set covers about 600, 500, and 450 data points per Mie fluid for the self-diffusivity, shear viscosity, and thermal conductivity, respectively. The phase space distribution for each transport property is shown in Figure 1. The database was purposely populated close to the phase envelope for temperatures up to 2.0. Higher-temperature isotherms up to 10.0 are sparsely covered. The density distribution of the database is evenly distributed except at high densities and low temperatures. This unpopulated area at high densities corresponds to the region where Mie fluid freezes.^21^

**Figure 2:**
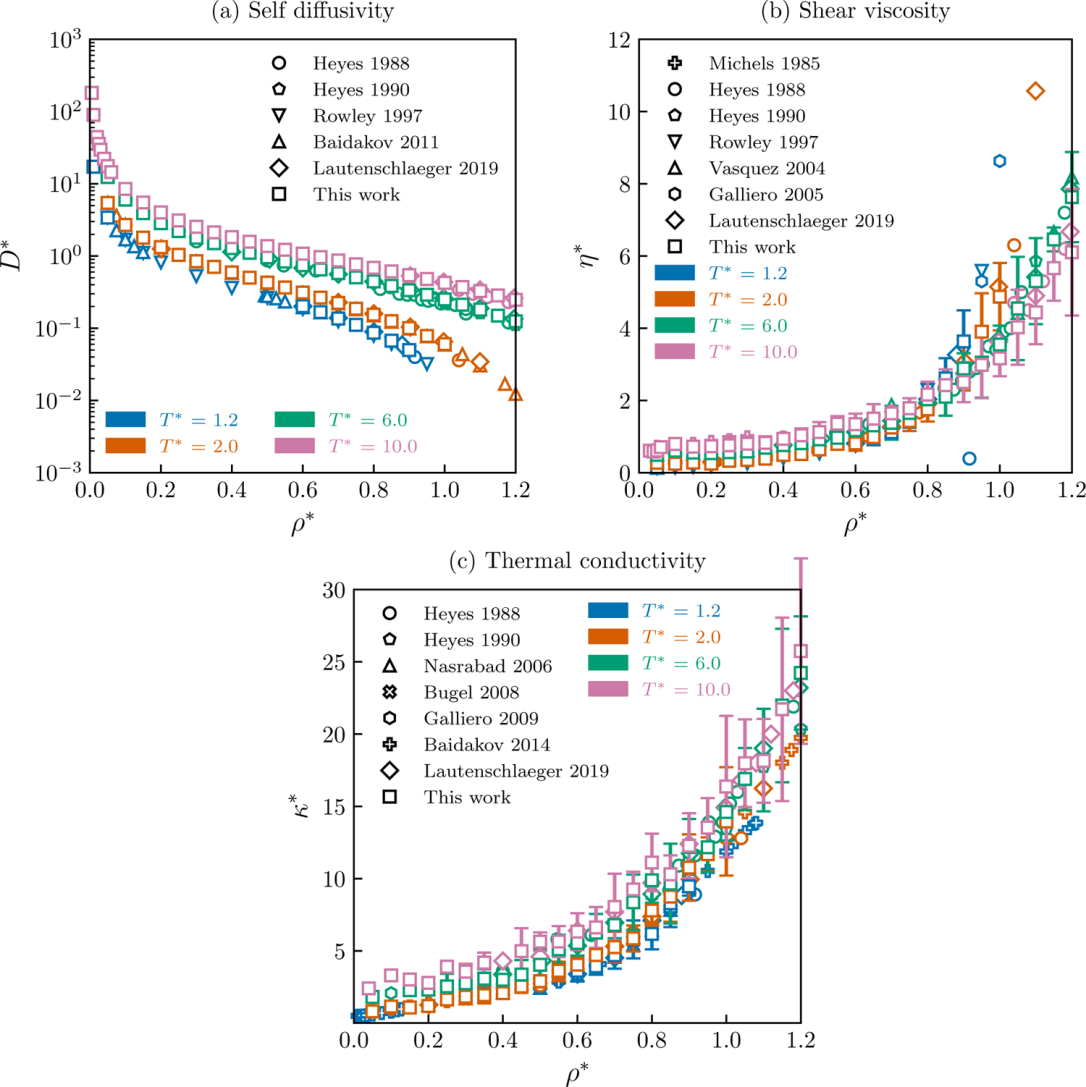
Transport simulation data of the Lennard-Jones fluid (λ_r_ = 12 and λ_a_ = 6). Blue symbols: T* = 1.2, orange symbols: T* = 2.0, green symbols: T* = 6.0, pink symbols: T* = 10.0. (a) Self-diffusivity. Published data: circles,^81^ pentagons,^82^ inverted triangles,^83^ triangles,^84^ diamonds.^35^ This work: squares. (b) Shear viscosity. Published data: plus symbol,^85^ circles,^81^ pentagons,^82^ inverted triangles,^83^ triangles,^86^ hexagons,^87^ diamonds.^35^ This work: squares. (c) Thermal conductivity. Published data: circles,^81^ pentagons,^82^ triangles,^88^ “X” symbol,^89^ hexagons,^90^ plus symbol,^91^ diamonds.^35^ This work: squares. Errors bars refer to the 95% confidence interval.

The database is assessed against published transport properties for the Lennard-Jones fluid (λ_r_ = 12 and λ_a_ = 6), previously compiled by Lautenschlaeger and Hasse.^35^ In Figure 2, the uncorrected self-diffusivity, shear viscosity, and thermal conductivity obtained in this work are compared against published transport properties at four different temperatures, namely, T* = 1.2, 2.0, 6.0, and 10.0. From the figure, it can be observed that the computed transport properties are in good agreement with the published Lennard-Jones data. By comparing the shear viscosity and thermal conductivity results to the self-diffusivity results, it can be noticed that the former exhibit higher deviations, especially at high temperatures and densities. This problem has been well reported in the literature and is thought to be a consequence that different trajectories can lead to significantly different transport properties results.^54,80^ A further source of deviations for the shear viscosity and thermal conductivity comes from the numerical integration of eqs 5 and 6 using Simpsons’s rule.^55^ In contrast, this integral is analytically available from the step integration algorithm for the self-diffusivity (see eq 3b).

### 3.2 Physically Inspired Modeling of Transport Properties

As mentioned in Section 2.2, transport properties (γ*) can be modeled as a function of density and temperature (γ* = ANN(α_vdW_, ρ*, T*)) or using entropy scaling (γ̃* = ANN(α_vdW_, −S^*,res^)). As pointed out previously by us,^30^ it is also convenient to consider an inverse of the temperature functionality (γ* = ANN(α_vdW_, ρ*, 1/T*)). By using the inverse of the temperature, the temperature scale shrinks, easing the interpolation efforts of the ANN. Additionally, it is important to consider the numerically more convenient and physics-informed outputs of the data-driven model.^30,92^ Additionally, the inverse of the temperature dependence is found in thermodynamic scaling,^93^ in which a transport property is a function of the (ρ*)^δ^/T* ratio, where δ is a scaling exponent. This dependence can be justified from isomorph theory.^39^

**Figure 3:**
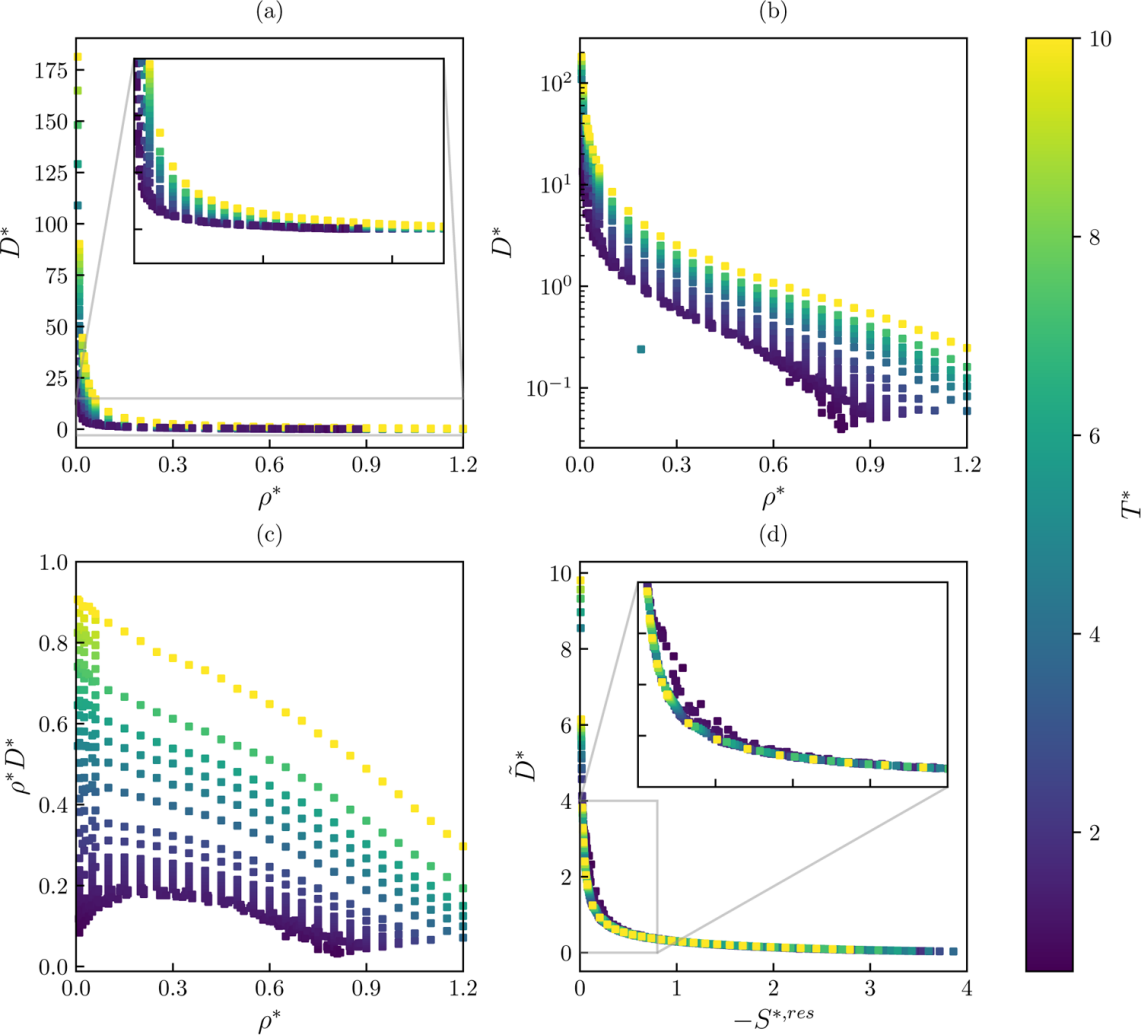
Self-diffusivity of the Lennard-Jones fluid (λ_r_ = 12 and λ_a_ = 6). (a) D*—linear scale, (b) D*—semilog scale, (c) ρ*D*—linear scale, (d) D̃*, reduced self-diffusivity eq 10a—linear scale. Color map refers to the temperature.

In Figure 3, different representations of the uncorrected self-diffusivity of the Lennard-Jones fluid are shown. As can be seen from Figure 3a, modeling the self-diffusivity (D*) with a linear scale results in a difficult task. The value of the transport property considerably increases at low densities. This scale also makes it challenging to differentiate results at moderate to high densities at different temperatures. This numerical issue can be solved using a semilog scale, as shown in Figure 3b. Similarly, modeling ρ*D* instead of the self-diffusivity itself is numerically convenient, as the values are easily separable in the ρ*–T* space, and the results are regularized, i.e., they are bounded between a narrow range (0 and 1 for the available database). Finally, from Figure 3d, it can be observed that the scaled self-diffusivity (eq 10a) is conveniently described as a function of the residual entropy. However, it has to be noticed that there is a range (zoomed region) in which the residual entropy can predict more than one self-diffusivity value. This behavior has also been observed in other entropy scaling studies of the self-diffusivity^41,42^ and will likely produce conflicting regions when training a data-driven model. At this point, several possible outputs of the data-driven model can be considered to model the self-diffusivity. A similar analysis is available for the shear viscosity and thermal conductivity in the Supporting Information.

Additionally, to regularize the transport properties data, the training can be improved by imposing physical-inspired constraints on the ANNs’ model. For example, the self-diffusivity is expected to be a decreasing function with respect to the density. The thermal conductivity, on the other hand, is a monotonically increasing function with respect to the density, except in the vicinity of the critical point where a maximum is expected. This critical enhancement has been experimentally observed in real molecules,^94−96^ and it is linked to the heat capacity maximum on the same region.^97^ Similarly, the shear viscosity exhibits a critical enhancement in a narrow region close to the critical point. This effect can be safely neglected for practical applications.^43^ That being said, the shear viscosity is expected to be an increasing function of the density near the critical point and a monotonically increasing function of the density for supercritical temperatures.^96^ Even though one could expect these behaviors to be discovered by a machine learning model, in this work, if needed, we prefer to enforce it using a physically informed approach.^98^ For example, the following penalty functions could be added to the loss function (eq 14) to guarantee the correct shear viscosity density derivatives:
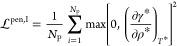
16a
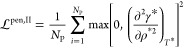
16bThe first penalty function, eq 16a, enforces that the shear viscosity is an increasing function with respect to the density (i.e., (∂γ*/∂ρ*)_T*_ > 0). The second penalty function, eq 16b, enforces convexity of the ANN model (i.e., (∂^2^γ*/∂ρ^*2^)_T*_ > 0).

Before training the data-driven models, a clarification has to be made when modeling the self-diffusivity. As mentioned in Section 2.1, the self-diffusivity should be corrected for finite-size effects. The correction term (eq 8) involves using the shear viscosity. One problem here is that the sizes of the databases for the self-diffusivity do not exactly match, meaning that not all of the self-diffusivity values could be corrected. Second, as pointed out before, the shear viscosity results exhibit higher deviations. This noise might contaminate the self-diffusivity results. For these reasons, we have deliberately chosen to model uncorrected self-diffusivity. The output of this self-diffusivity model can be corrected afterward using the produced data-driven model for the shear viscosity as follows:
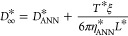
17In eq 17, ξ = 2.837298 and , where *N* equals to the number of particles (4096 for this work) and *D*_ANN_^*^ and η_ANN_^*^ are the outputs of the artificial network models for the self-diffusivity and shear viscosity, respectively.

The ANN’s architecture to model each transport property was optimized using Optuna.^79^ The obtained architectures are as follows:

- Self-diffusivity: 3 hidden layers with 20 neurons each using the tanh activation function.
- Shear viscosity: 4 hidden layers with 20 neurons each using the tanh activation function.
- Thermal conductivity: 3 hidden layers with 30 neurons each using the tanh activation function.

The output layer can use either the linear (*f*(*x*) = *x*) or softplus activation function (*f*(*x*) = ln (1 + exp(*x*)), depending on the output feature. All of the ANNs were trained using the Adam optimizer^74^ with a learning rate (α) of 10^–3^ and a maximum of 1000 epochs. Further details about the hyperparameter optimization results can be found in the Supporting Information.

The results for the different trained ANNs are shown in Table 2. For the case of self-diffusivity, all modeling approaches use a linear activation function for the output layer except the entropy scaling, which uses the softplus activation function. The results from modeling ln D* and ρ*D* exhibit lower deviations. This result is a direct consequence of facilitating the interpolating efforts of the ANN, as shown in Figure 3b,c. It can also be noticed that, generally, using the inverse of the temperature as an input feature produces models with lower deviations. For the self-diffusivity, as expected, the entropy scaling approach produces higher deviations, as observed in Figure 3d, as there are conflicting regions in which a single value of residual entropy can lead to multiple self-diffusivities. It can also be observed that the ANN models present similar deviations in the training and testing data sets, implying negligible overfitting. Finally, for this property, the best-performing model is ρ*D* = ANN(α_vdW_, ρ*, 1/T*).

**Table 2:** Transport Properties: Relative Absolute Average Deviations (% AAD) from Models Based on Artificial Neural Networks (ANNs)^h^

		% AAD transport property^a^^,^^b^	
transport property	model	train	test
self-diffusivity (D*)^c^	D* = ANN(α_vdW_, ρ*, T*)^f^	8.43	8.74
	D* = ANN(α_vdW_, ρ*, 1/T*)^f^	5.84	6.15
	ln D* = ANN(α_vdW_, ρ*, T*)^f^	0.72	0.72
	ln D* = ANN(α_vdW_, ρ*, 1/T*)^f^	0.89	0.91
	ρ*D* = ANN(α_vdW_, ρ*, T*)^f^	1.00	0.98
	ρ*D* = ANN(α_vdW_, ρ*, 1/T*)^f^	**0.55**	**0.56**
	D̃* = ANN(α_vdW_, −S^*,res^)^g^	6.15	6.30
shear viscosity (η*)^d^	η* = ANN(α_vdW_, ρ*, T*)^g^	5.80	5.94
	η* = ANN(α_vdW_, ρ*, 1/T*)^g^	5.76	5.90
	ln η* **= ANN(**α_vdW_, ρ*, T*)^f^	5.61	5.55
	ln η* = ANN(α_vdW_, ρ*, 1/T*)	**5.61**	**5.54**
	η̃* = ANN(α_vdW_, −S^*,res^)^g^	6.14	6.22
thermal conductivity (κ*)^e^	κ* = ANN(α_vdW_, ρ*, T*)^g^	6.15	6.36
	κ* = ANN(α_vdW_, ρ*, 1/T*)^g^	6.38	6.60
	ln κ* = ANN(α_vdW_, ρ*, T*)^f^	5.94	6.22
	lnκ* = ANN(α_vdW_, ρ*, 1/T*)^f^	**5.95**	**6.12**
	κ̃* = ANN(α_vdW_, −S^*,res^)^g^	6.77	6.91

a .

b The % AAD is computed for the given transport property (D*, η*, or κ*).

c The ANN consists of 3 hidden layers with 20 neurons, each using the tanh activation function.

d The ANN consists of 4 hidden layers with 20 neurons, each using the tanh activation function.

e The ANN consists of 3 hidden layers with 30 neurons, each using the tanh activation function.

f The output layer uses the linear activation function.

g The output layer uses the softplus activation function.

h Bold lettering denotes the best-performing ANNs.

For the case of the shear viscosity and thermal conductivity, the hyperparameter optimization detected an exponential dependency of the property with respect to the inputs, prompting us to use the softplus activation function for the output layer when modeling the transport property (γ*) or the scaled value (γ̃*). Additionally, the use of this activation function ensures that the output is always positive. On the other hand, the linear activation function is used when modeling the logarithm of the transport property (ln γ*). For these two transport properties, the deviations of the models are higher than for the self-diffusivity. We attribute these higher deviations to the inherent noisiness of the data.

In general, it is observed that using entropy scaling leads to higher deviations. Some of these deviations seem to be inherent of the underlying premise. Modeling scaled transport properties based on the residual entropy can be justified from the isomorph theory.^40^ In this theory, it is concluded that only the transport properties of a fluid interacting through an inverse power law (i.e., ) are a monovariate function of the residual entropy.^43^ For other fluids, such as the Mie fluid, the conditions are only met for dense (liquid) states,^38,42^ and a divergence is observed at low densities. There exist workarounds in the literature to deal with low-density regions either by using a modified scaling,^41^ using a modified input variable based on the residual entropy^44^ or by modeling the “residual” transport property.^99^ However, it has also been argued that the use of these workarounds negatively impacts the results of the liquid phase.^42^ We conclude here that for this particular application, using entropy scaling over the entire density space is impractical.

A direct correlation using density and inverse of the temperature provides the best fit for both the training and testing data sets. Moreover, using the logarithm of the property also implies a lower deviation. Using a semilog scale makes the training outputs to be in the same order of magnitude, which prevents a loss function based on the mean squared error from being biased to fit only values with a high magnitude. That being said, the best-performing models for the shear viscosity and thermal conductivity are ln η* = ANN(α_vdW_, ρ*, 1/T*) and ln κ* = ANN(α_vdW_, ρ*, 1/T*), respectively.

**Figure 4:**
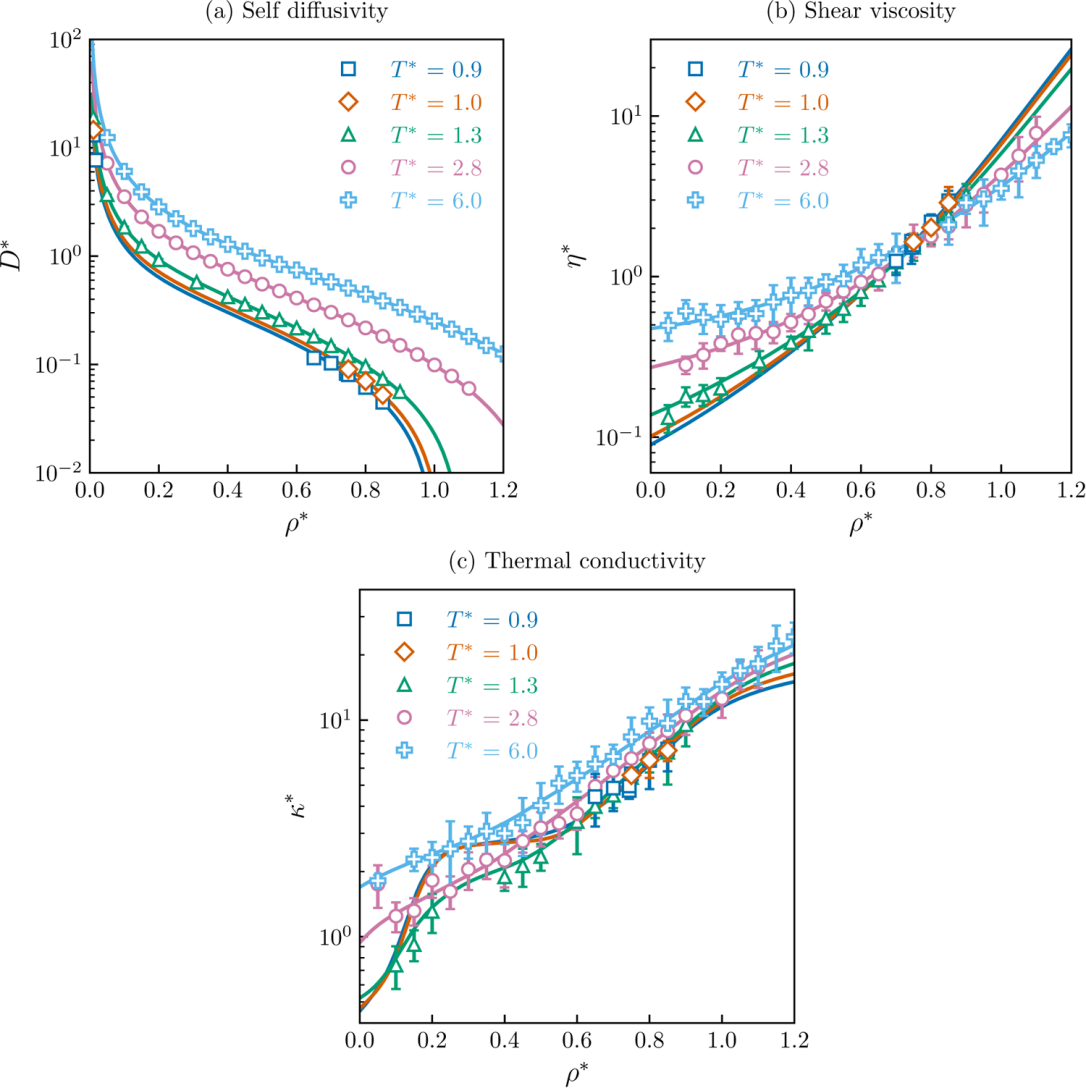
Transport properties isotherms for the Lennard-Jones fluid (λ_r_ = 12 and λ_a_ = 6). Molecular dynamics data: Blue squares: T* = 0.9, orange diamonds: T* = 1.0, green triangles: T* = 1.3, pink circles: T* = 2.8, and sky blue plus sign: T* = 6.0. Solid lines: (a) Self-diffusivity (ρ*D* = ANN(α_vdW_, ρ*, 1/T*)), (b) shear viscosity (ln η* = ANN(α_vdW_, ρ*, 1/T*), trained with penalty functions (eqs 16a and 16b), and (c) thermal conductivity (ln κ* = ANN(α_vdW_, ρ*, 1/T*)).

Even though it is well known that machine learning models can achieve high accuracy in describing thermophysical properties,^30,46,92,100^ it is also important to consider the consistency and physicality of the predictions.^101^ In Figure 4, five predicted isotherms using the trained ANNs are shown for each transport property of the Lennard-Jones fluid (λ_r_ = 12 and λ_a_ = 6). The isotherms are computed with the best-performing models, as shown in Table 2. The isotherms include two subcritical isotherms (T* = 0.9 and 1.0), one near-critical isotherm (T* = 1.3), and two supercritical isotherms (T* = 2.8 and 6.0). Transport properties isotherms for other selected Mie fluids can be found in the Supporting Information. As expected from the magnitude of the deviations, the model for self-diffusivity, shown in Figure 4a, follows a well-behaved trend on the entire density and temperature space. This behavior is even observed in regions with no data available, such as the region that lies inside the vapor–liquid phase envelope. For the case of the shear viscosity, the best-trained model, as reported in Table 2, produces oscillatory and physically incorrect isotherms. The reported oscillatory results can be found in the Supporting Information. This issue is addressed by employing the penalty functions (eqs 16a and 16b). It has to be considered that the training considering derivative information tends to be numerically unstable, as even small changes in the weights and biases can lead to significant changes on the first and second derivatives.^30^ For this reason, the learning parameter (α) is reduced to 10^–5^. In order to overcome the slow training rate, the maximum number of epochs is increased to 2000. This physically corrected viscosity model predicts the training and testing data set with an % AAD of 5.73 and 5.67, respectively. This slight deviation increase is a price worth paying, as shown in Figure 4b; the produced model is nonoscillatory and follows an expected physical behavior. Finally, in Figure 4c, the computed five isotherms for the thermal conductivity of the Lennard-Jones fluid are shown. The predicted isotherms are smooth and nonoscillatory. Additionally, for this transport property, the expected maximum in the vicinity of the critical density for the near-critical and subcritical isotherms is predicted by the ANN model.

**Figure 5:**
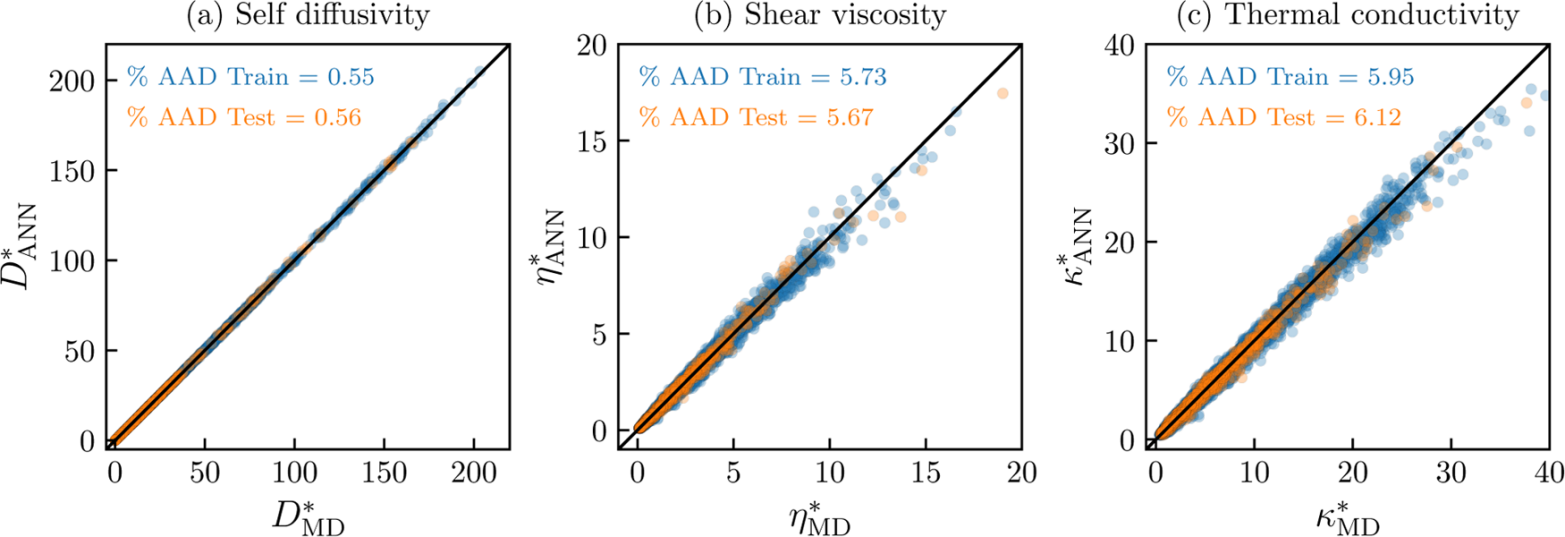
Parity plots for the trained transport properties of ANNs models for the Mie fluid with respect to molecular dynamics data. Training data: blue circles, test data: orange circles. (a) Self-diffusivity (ρ*D* = ANN(α_vdW_, ρ*, 1/T*)), (b) shear viscosity (ln η* = ANN(α_vdW_, ρ*, 1/T*), trained with penalty functions (eqs 16a and 16b), and (c) thermal conductivity (ln κ* = ANN(α_vdW_, ρ*, 1/T*)).

**Figure 6:**
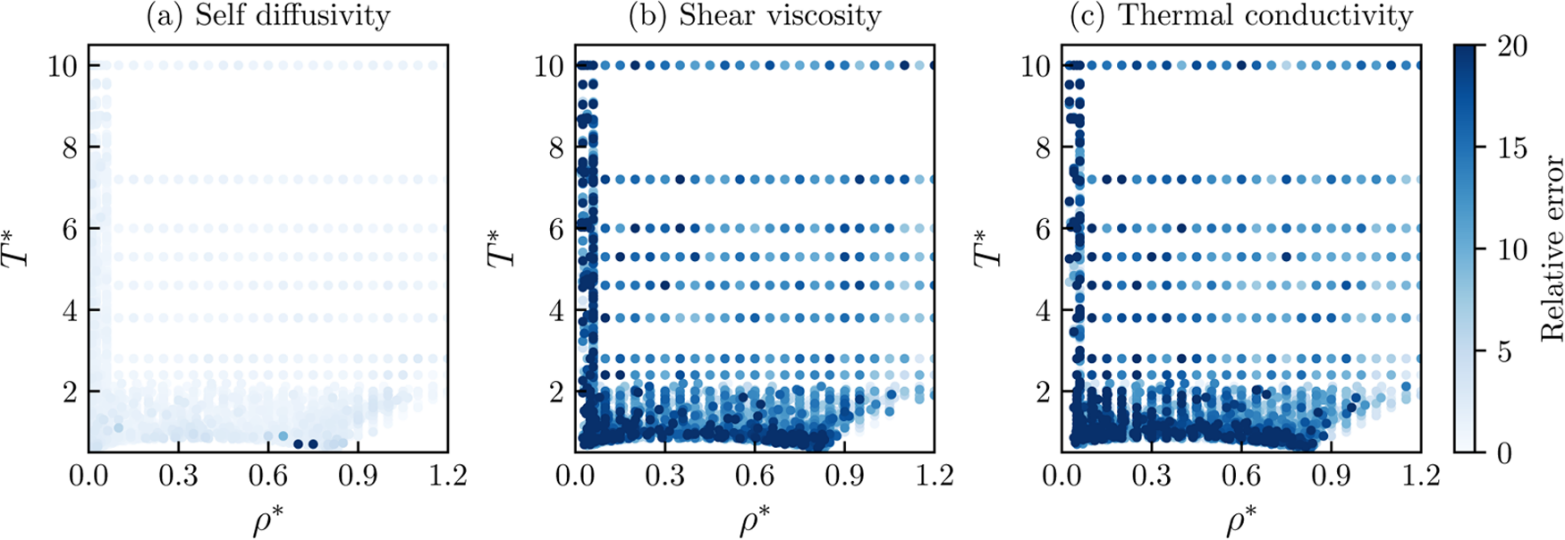
Relative error distribution for the trained transport properties of ANNs models for the Mie fluid with respect to molecular dynamics data. Darker symbols refer to a higher deviation. (a) Self-diffusivity (ρ*D* = ANN(α_vdW_, ρ*, 1/T*)), (b) shear viscosity (ln η* = ANN(α_vdW_, ρ*, 1/T*), trained with penalty functions (eqs 16a and 16b), and (c) thermal conductivity (ln κ* = ANN(α_vdW_, ρ*, 1/T*)).

The overall performance of the best transport properties models is also assessed in Figures 5 and 6. In Figure 5, the parity plots of each transport property are shown for the training and test data sets. In Figure 5a, the results for the self-diffusivity show that almost all data points lie on the diagonal for a wide range of values. For the case of the shear viscosity and thermal conductivity, Figure 5b,c, it can also be observed that the points lie within the diagonal. As discussed previously, these data points are inherently noisier, and a more noticeable dispersion was expected. For the case of the three transport properties, it can be seen that the ANN’s model predicts the unseen test data similarly to the training data, disregarding the possible overfitting of the models.

In Figure 6, possible trends for the relative deviations can be identified in the density–temperature space. Darker symbols refer to data points the ANN’s models describe with a higher deviation. From Figure 6a, it can be seen that most of the self-diffusivity data points are correctly described except for a few exceptions at low temperatures. These data points are likely to be close to (or inside) the phase envelope and could potentially be incorrect. For the case of the shear viscosity and thermal conductivity, Figure 6b,c, it can be noticed that higher deviation points are found in the low-density region and close to the region where vapor–liquid phase envelope should lie. First, higher relative deviations at the low-density regions are also an artifact of calculating the relative deviation of a considerably small number, and no special treatment has been done to deal with the low-density region (ρ* → 0). Additionally, higher relative deviations were expected for these transport properties due to the noisiness of the molecular simulation data.

**Figure 7:**
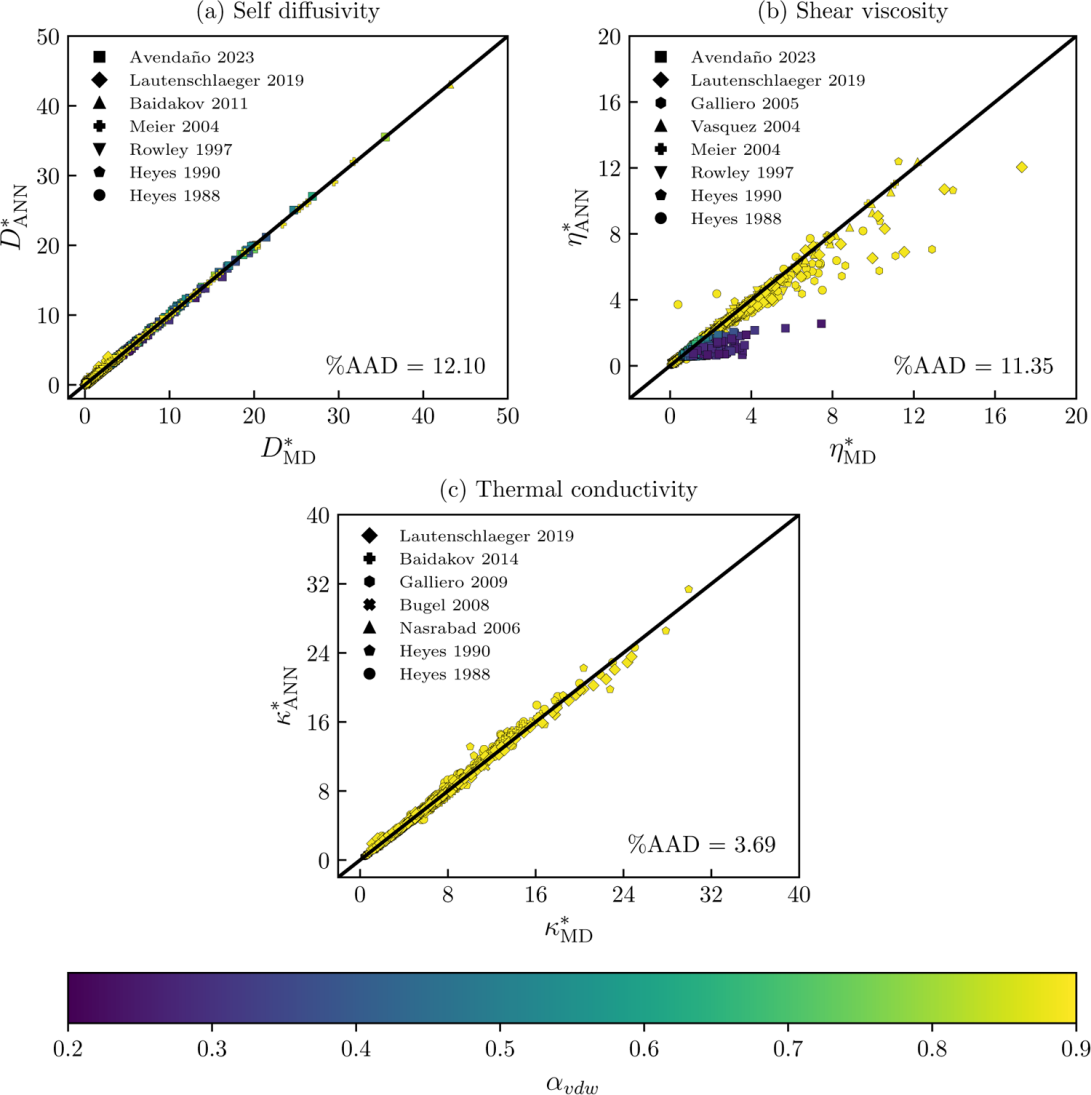
Parity plots for the trained transport properties of ANNs models for the Mie fluid with respect to published molecular dynamics data. (a) Self-diffusivity (ρ*D* = ANN(α_vdW_, ρ*, 1/T*)), (b) shear viscosity (ln η* = ANN(α_vdW_, ρ*, 1/T*), trained with penalty functions (eqs 16a and 16b), and (c) thermal conductivity (ln κ* = ANN(α_vdW_, ρ*, 1/T*)). Published data: (a) squares,^46^ diamond,^35^ triangle,^84^ plus sign,^105^ upside triangle,^83^ pentagon,^82^ and circle.^81^ (b) Shear viscosity: (a) squares,^46^ diamond,^35^ hexagon,^87^ triangle,^86^ plus sign,^106^ upside triangle,^83^ pentagon,^82^ and circle.^81^ (c) Thermal conductivity: diamond,^35^ plus sign,^91^ hexagon,^90^ X sign,^89^ triangle,^88^ pentagon,^82^ and circle.^81^ Color map refers to the α_vdW_ parameter of the Mie fluid (eq 9). Yellow symbols refer to the Lennard-Jones fluid (α_vdW_ = 0.88).

The best-performing transport properties models have also been tested on published data. This includes the data collected by Lautenschlaeger and Hasse^35^ for the Lennard-Jones fluid and the recent data for self-diffusivity and shear viscosity for selected Mie fluids obtained by Ŝlepaviĉius et al.^46^ It has to be considered that even though the same underlying interaction potential is being used, the molecular dynamics simulations differ in the cutoff radius and the number of particles; hence, the results from the ANN’s models are only partially comparable to the published data. Nevertheless, they still allow assessing the behavior of the trained models in untrained conditions. The parity plots for this data are shown in Figure 7. The symbols are colored based on the α_vdW_ parameter (eq 9). As a reference, this parameter equals 0.88 for the Lennard-Jones fluid (yellow symbols in the figure). From Figure 7a, it can be observed that most self-diffusivity data points are correctly described. The global relative deviation for this property equals 12.10%. The main source of discrepancy is attributed to data points at very high densities (ρ* > 1.2),^82^ at metastable conditions,^84^ and small α_vdW_ values,^46^ which are far from the training range (0.53 < α_vdW_ < 1.47).

The parity plot for the shear viscosity is shown in Figure 7b. For this transport property, the model fails to extrapolate at very low α_vdW_ values,^46^ which are indicated by dark blue colors. We noted that these values of the α_vdW_ are obtained for highly attractive exponents; this is λ_a_ in the order of 10 to 14. It has been shown that some extreme combinations of the repulsive–attractive exponents of the Mie potential may result in the breaking down of the corresponding states principle.^102^ Hence, we recommend using the developed models only for λ_r_ – 6 Mie fluids. Additionally, it is plausible that the conformality implied by eq 9 does not hold for transport properties. For the case of the discrepancies between the published Lennard-Jones data, we found that the main discrepancies are for data points that lie inside the vapor–liquid phase envelope. This is a direct consequence of comparing simulation results with different cutoff radii (e.g., Lautenschlaeger and Hasse^35^ use shifted potential with *r*_c_ = 2.5σ). The cutoff radius modifies the unstable region of the Lennard-Jones fluid.^103,104^

Finally, in Figure 7c, the predicted thermal conductivities are compared to the published data. For this transport property, only data for the Lennard-Jones fluid is compared. As can be observed from the figure, most data points are correctly described. The global relative deviation for this property is 3.69%. Under the inspection of the relative residuals, the more significant deviations are found for data points inside the unstable region for the Lennard-Jones fluid with the cutoff radius used in this work (*r*_c_ = 5σ). The errors in that region can be considerable, as shown in Figure 4c; the ANN model predicts a maximum for subcritical temperatures near the critical density.

Until this point, the developed data-driven models do not consider any special treatment to handle the dilute gas state (ρ* → 0), and their transport property prediction is an extrapolation of the model. However, this low-density region can be well described from kinetic theory,^107,108^ where a transport property is related to an expansion based on collision integrals. For the explored temperature conditions in this work (T* ≤ 10), the first-order approximation is enough to describe a transport property within a 1% error.^107^ In this case, the dilute gas transport properties are obtained as follows.
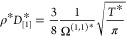
18a
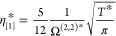
18b
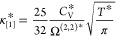
18cIn eqs 18a–18c, D*_[1]_, η*_[1]_, and κ*_[1]_ are the first-order approximations for the self-diffusivity, shear viscosity, and thermal conductivity, respectively. Ω^(l,s)*^ refers to a collision integral,^107,108^ and *C*_V_* = *C*_V_/*R* is the dimensionless isochoric heat capacity, equal to 1.5 for monatomic molecules at zero density. The calculation of the collision integral is not trivial; however, values of Ω^(1,1)*^ and Ω^(2,2)*^ have been computed and correlated for λ_r_ – 6 Mie fluids with λ_r_ ∈ [8, ∞] and T* ∈ [0.4, 200] by Fokin et al.^109^ See the Supporting Information for further information.

Equations 18a–18c and the correlations of Ω^(1,1)*^ and Ω^(2,2)*^ developed by Fokin et al.^109^ can be used to develop data-driven models that exactly fulfill the first-order transport property approximation from kinetic theory for λ_r_ – 6 Mie fluids. This approach is in line with the fact that the previously developed models fail to predict the transport properties of highly attractive Mie fluids,^46^ and these should be valid only for λ_r_ – 6 Mie fluids. In this case, the data-driven models are formulated as follows.
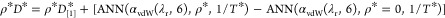
19a
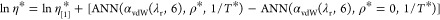
19b
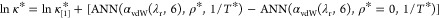
19cThese data-driven models exactly fulfill the first-order kinetic theory approximation at zero density and have been trained to learn only the residual contribution to a transport property. These models are only valid for λ_r_ – 6 Mie fluids with λ_r_ ≥ 8.

**Figure 8:**
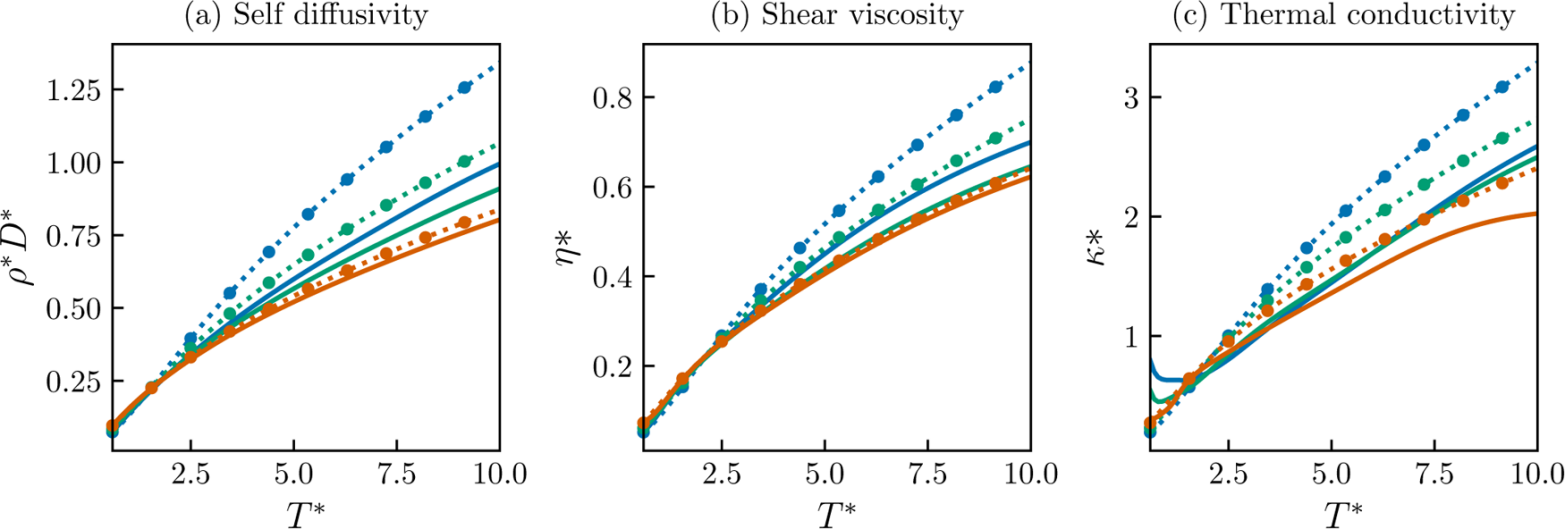
Transport properties at zero density for selected λ_r_ – 6 Mie fluids. (a) Self-diffusivity, (b) shear viscosity, and (c) thermal conductivity. Circles: Kinetic theory results (eqs 18a–18c), solid lines: ANN models (eq 112a), dotted lines: ANN dilute gas models (eqs 19a–19c). Blue: λ_r_ = 8, green: λ_r_ = 12, orange: λ_r_ = 24.

The training and test % AAD for the trained self-diffusivity (eq 19a), shear viscosity (eq 19b), and thermal conductivity (eq 19c) residual models are 0.70/0.68, 5.86/6.07, and 6.38/6.57, respectively. These deviations are slightly higher than the ones found in Table 2; however, these models correctly describe the dilute gas limit, as shown in Figure 8. Similar figures to Figures 4 and 5 for these residual models can be found in the Supporting Information. We recommend using these models when dealing with λ_r_ – 6 Mie fluids.

The training and testing data sets and the trained data-driven transport properties models produced in this work are freely available on GitHub.

## 4 Conclusions

In this work, the self-diffusivity (D*), shear viscosity (η*), and thermal conductivity (κ*) of the Mie fluid have been computed and modeled. The transport properties of the Mie fluid have been modeled using artificial neural networks. Two main modeling approaches were tested: a semiempirical formulation based on entropy scaling or an empirical formulation based on density and temperature as input variables. For the former, it was found that a unique formulation based on entropy scaling does not lead to satisfactory results over the entire density range, with issues mainly associated with a divergent and incorrect scaling of the transport properties at low densities. It has been found that regularizing the data, e.g., modeling ρ*D* instead of D*, ln η* instead of η*, and ln κ* instead of κ*, as well as using the inverse of the temperature as input feature, helps to ease the interpolation efforts of the ANNs. The trained ANNs can model seen and unseen data over a wide range of density temperatures. Moreover, they also predict the expected physical behavior. For example, the self-diffusivity model suggests that the diffusivity is a smooth decreasing function of density and an increasing function of temperature. The ANN model for thermal conductivity exhibits the well-reported critical enhancement. With this approach, a wavy and nonphysical model was obtained for the shear viscosity. This behavior is a clear case in which a model with low deviations is obtained but with incorrect physical behavior. This problem was successfully addressed by performing a physically informed training in which the derivatives of the model are forced to be positive, producing an ANN model that is an increasing function with respect to the density.

We suggest using the ANN models highlighted in bold in Table 2. The produced models perform well for unseen data within the training range, i.e., α_vdW_ ∈ [0.53, 1.47], ρ* ∈ [5·10^–3^, 1.2], and T* ∈ [0.6, 10.0]). However, the models may fail for unseen conditions, like an extremely attractive Mie fluid that produces low α_vdW_ values, highly dense conditions close to the solid state boundary, or at the zero density limit. As in general, with data-driven models, extrapolation should be avoided. Additionally, for the specific case of λ_r_ – 6 Mie fluids, we have developed ANN models that agree with the kinetic theory first-order approximation.

The ANN models can assess the performance of already published Mie fluid parameters for transport properties. Moreover, the ANN models can be used alongside an equation of state for the Mie fluid (like the FE-ANN EoS^30^ or SAFT-VR-Mie EoS^25^) to parametrize the Mie potential using equilibrium and transport properties simultaneously.

## Supporting Information Available

Numerical representation of the shear viscosity and thermal conductivity; hyperparameter optimization results for the ANNs; and transport properties results for selected Mie fluid (PDF)
The trained transport properties models and data generated in this article are available on the following GitHub repository: https://github.com/gustavochm/Transport-Properties-Mie-Fluid
