# Supplementary material for: Simulation and Data-Driven Modeling of the Transport Properties of the Mie Fluid
Source: J Phys Chem B. 2024 Jan 5;128(2):551–66. doi: 10.1021/acs.jpcb.3c06813 (PMC10801693; doi:10.1021/acs.jpcb.3c06813)
Supplement: Supplementary file 1 — jp3c06813_si_001.pdf [file jp3c06813_si_001.pdf]

Supporting Information: Simulation and Data-Driven Modeling of the Transport Properties of the Mie Fluid

Gustavo Chaparro and Erich A. Müller*

Department of Chemical Engineering, Sargent Centre for Process Systems Engineering, Imperial College London, South Kensington Campus, London SW7 2AZ, U.K.

E-mail: e.muller@imperial.ac.uk

1 Transport properties representation

Different numerical representations of the self diffusivity are presented and discussed in Section 3.2 of the main article. A similar analysis can be done for the shear viscosity (η^*) and thermal conductivity (κ^*). In the case of these transport properties, they can be represented using a linear scale, a semi-log scale, or using the following scaling needed for entropy scaling:

$$\tilde{\eta}^* = \frac{\eta^*}{(\rho^*)^{2/3}\sqrt{T^*}} \tag{10b}$$

$$\tilde{\kappa}^* = \frac{\kappa^*}{(\rho^*)^{2/3}\sqrt{T^*}} \tag{10c}$$

The different representations are shown in Figures S1 and S2 for the shear viscosity and thermal conductivity, respectively.

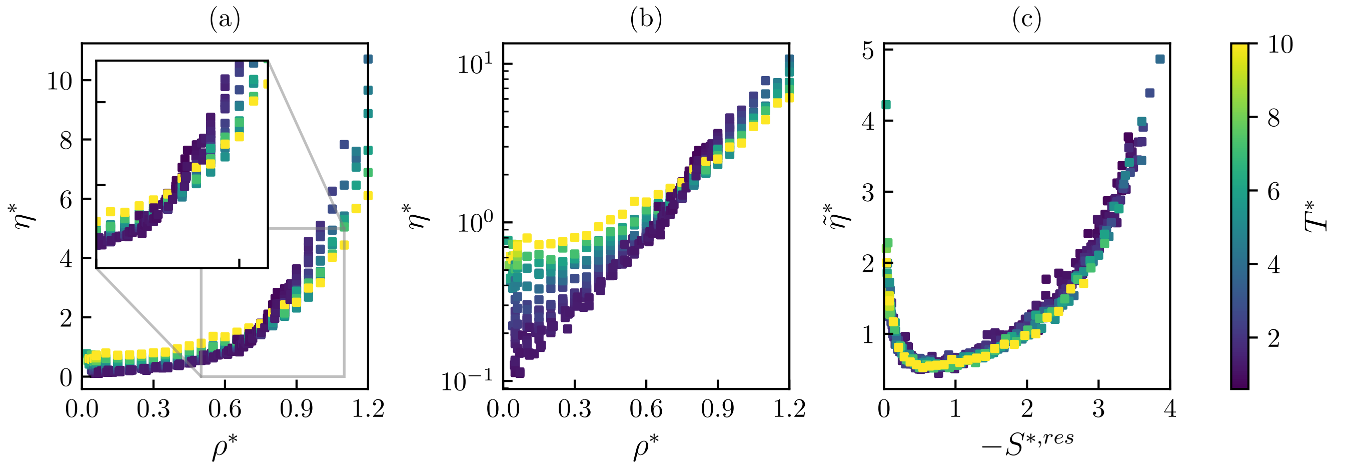

Figure S1: Shear viscosity of the Lennard-Jones fluid ($\lambda_r = 12$ and $\lambda_a = 6$). (a) η^* - linear scale, (b) η^* - semi-log scale, (c) $\tilde{\eta}^*$, reduced shear viscosity Eq. (10b) - linear scale. Colormap refers to the temperature.

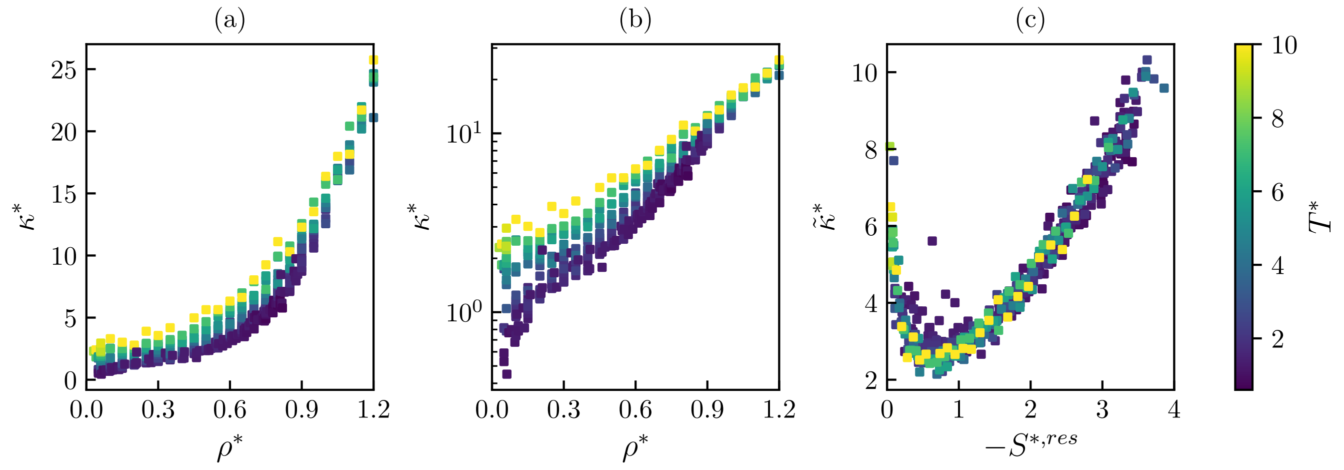

Figure S2: Thermal conductivity of the Lennard-Jones fluid ($\lambda_r = 12$ and $\lambda_a = 6$). (a) κ^* - linear scale, (b) κ^* - semi-log scale, (c) $\tilde{\kappa}^*$, reduced thermal conductivity Eq. (10c) - linear scale. Colormap refers to the temperature.

2 Hyperparameter Optimization of Artificial Neural Networks

The architecture of the artificial neural networks used to model the transport properties of the Mie fluid have been optimized using the `Optuna` package.[S1] The optimization procedure was constrained to the following practices to reduce the dimensionality problem.

- The hidden layers are restricted to having the same number of neurons.
- The hidden layers use the tanh activation function.
- The output layers can use either the linear or softplus activation function.
- The Adam optimizer is used for training.
- The batch size is set to 32.
- The maximum number of epochs is set to 1000.

The parallel plots, Figures S3, S4, S5, show the general trends that minimize the loss function for modeling the self diffusivity, shear viscosity, and thermal conductivity, respectively.

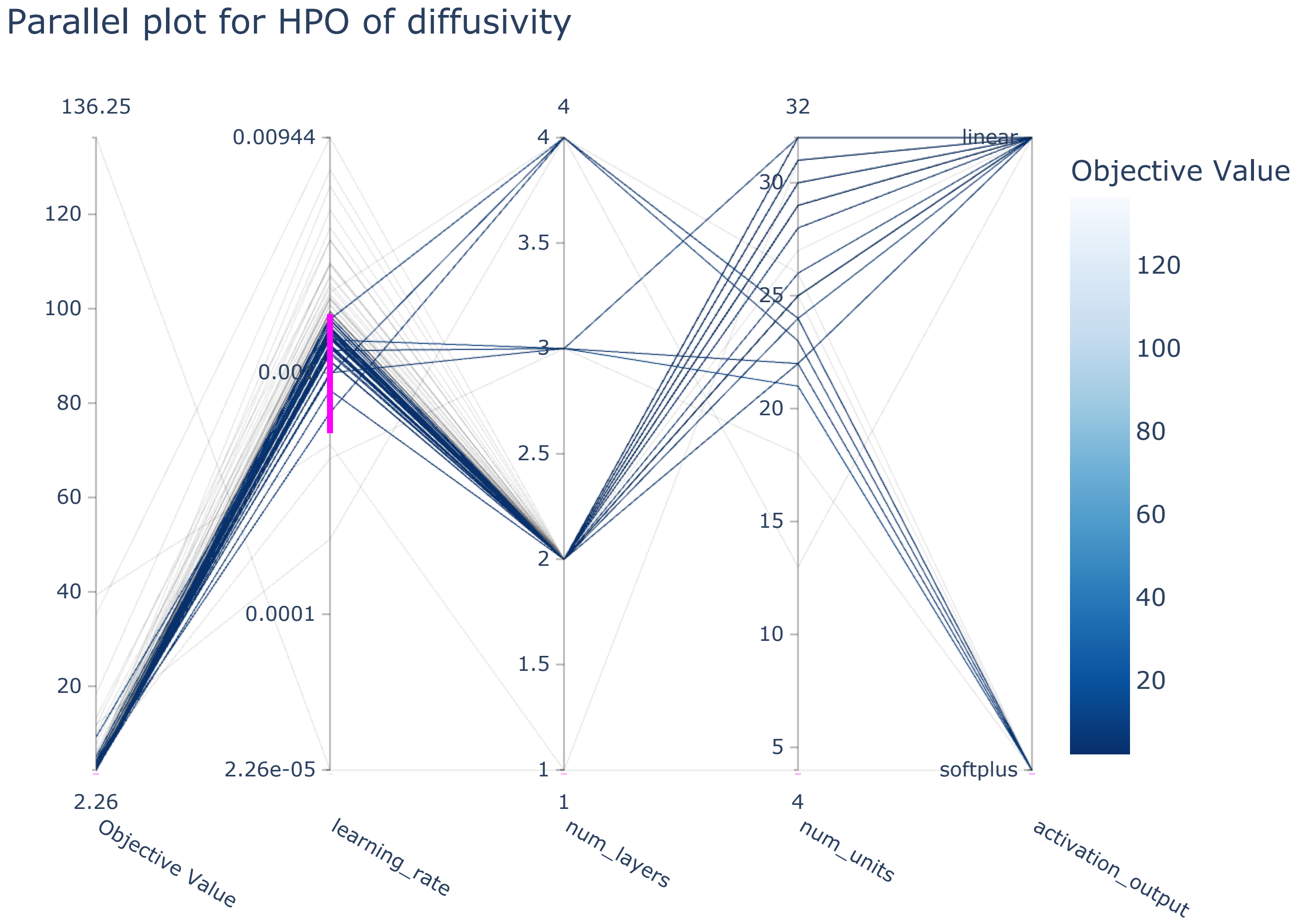

Figure S3: Parallel plot from hyperparameter optimization (HPO) of the modeling of self diffusivity of the Mie fluid using ANNs, i.e., $D^{*} = \mathrm{ANN}(\alpha, \rho^{*}, T^{*})$.

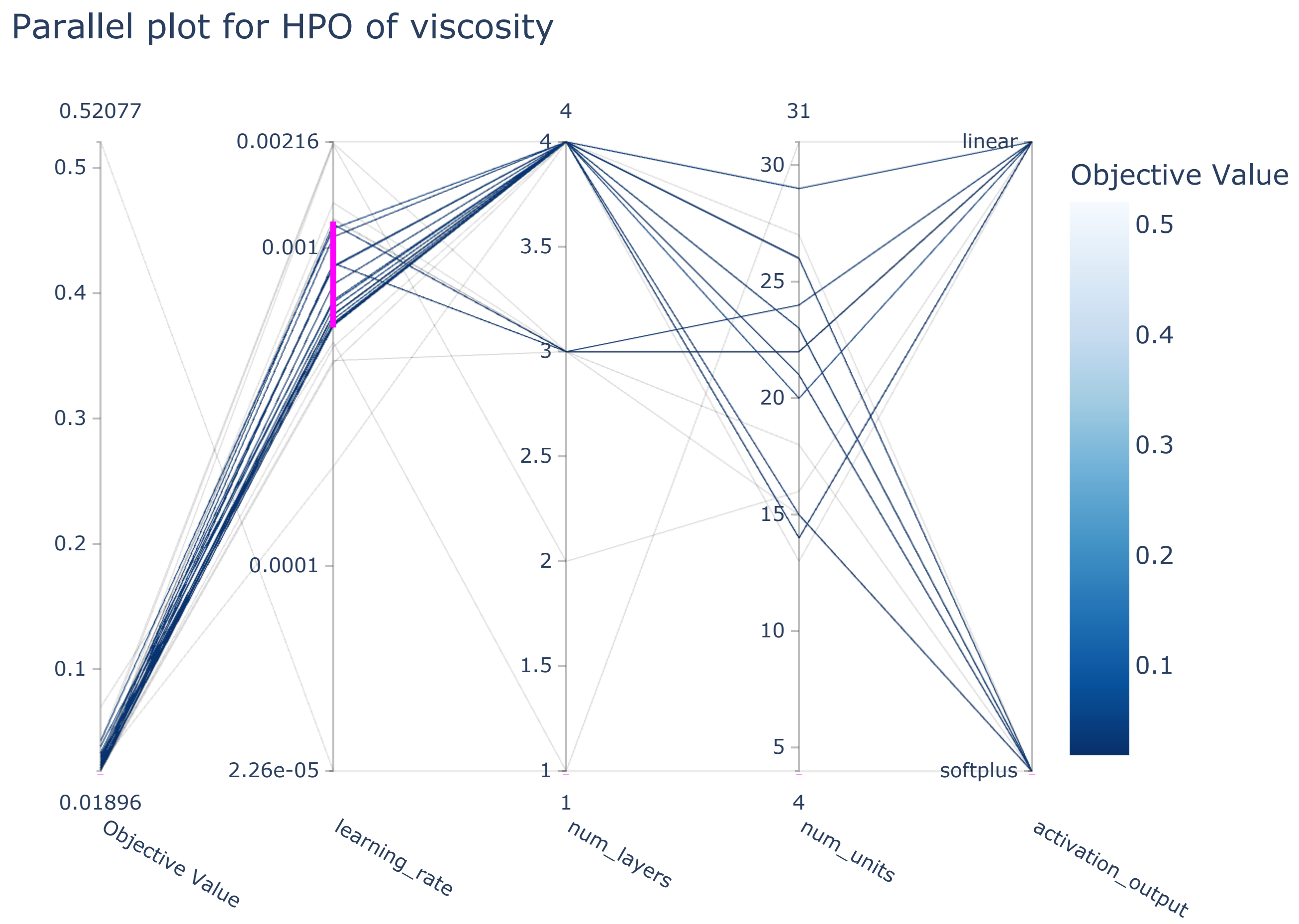

Figure S4: Parallel plot from hyperparameter optimization (HPO) of the modeling of shear viscosity of the Mie fluid using ANNs, i.e., $\eta^* = \mathrm{ANN}(\alpha, \rho^*, T^*)$.

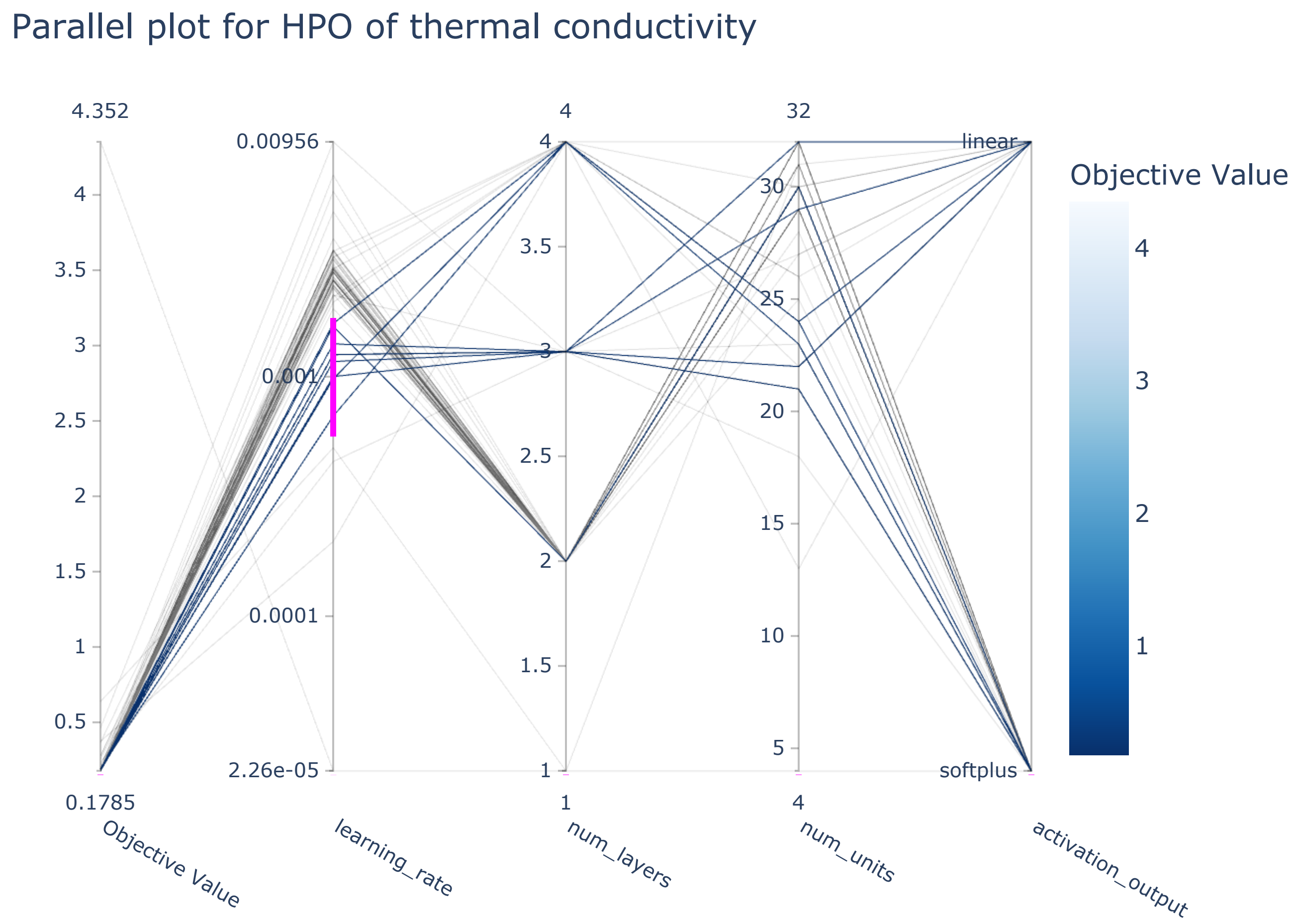

Figure S5: Parallel plot from hyperparameter optimization (HPO) of the modeling of thermal conductivity of the Mie fluid using ANNs, i.e., $\kappa^* = \mathrm{ANN}(\alpha, \rho^*, T^*)$.

3 Transport properties modeling of selected Mie fluids

3.1 $\lambda_r = 18$ and $\lambda_a = 6$

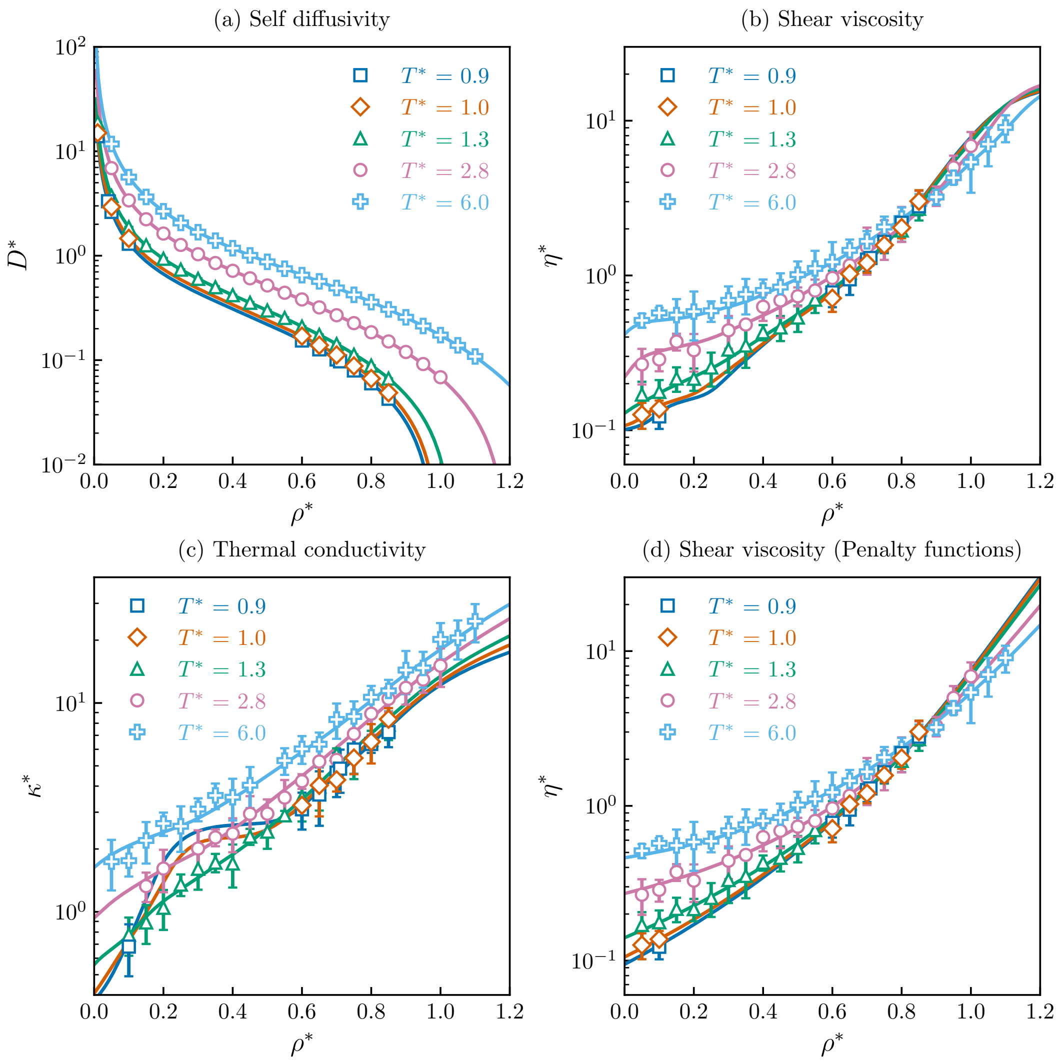

Figure S6: Transport properties isotherms for the Mie fluid ($\lambda_r = 18$ and $\lambda_a = 6$). Molecular dynamics data: Blue squares: $T^* = 0.9$, orange diamonds: $T^* = 1.0$, green triangles: $T^* = 1.3$, pink circles: $T^* = 2.8$ and sky blue plus sign: $T^* = 6.0$. Solid lines: (a) Self diffusivity ($\rho^* D^* = \text{ANN}(\alpha_{vdw}, \rho^*, 1/T^*)$), (b) Shear viscosity ($\ln \eta^* = \text{ANN}(\alpha_{vdw}, \rho^*, 1/T^*)$), (c) Thermal conductivity ($\ln \kappa^* = \text{ANN}(\alpha_{vdw}, \rho^*, 1/T^*)$), (d) Shear viscosity ($\ln \eta^* = \text{ANN}(\alpha_{vdw}, \rho^*, 1/T^*)$, trained with penalty functions (Eqs. (16)).

3.2 $\lambda_r = 24$ and $\lambda_a = 6$

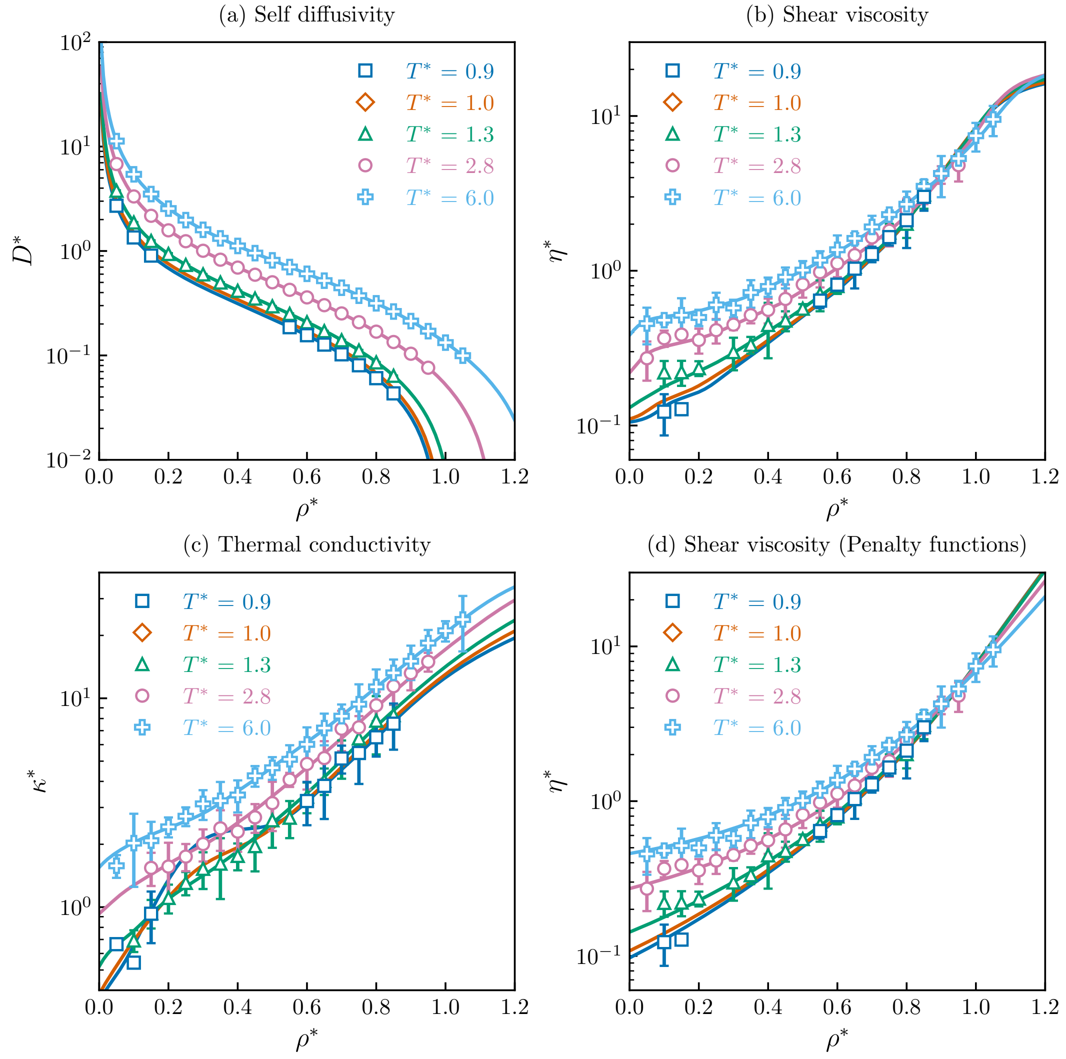

Figure S7: Transport properties isotherms for the Mie fluid ($\lambda_r = 24$ and $\lambda_a = 6$). Molecular dynamics data: Blue squares: $T^* = 0.9$, orange diamonds: $T^* = 1.0$, green triangles: $T^* = 1.3$, pink circles: $T^* = 2.8$ and sky blue plus sign: $T^* = 6.0$. Solid lines: (a) Self diffusivity ($\rho^* D^* = \text{ANN}(\alpha_{vdw}, \rho^*, 1/T^*)$), (b) Shear viscosity ($\ln \eta^* = \text{ANN}(\alpha_{vdw}, \rho^*, 1/T^*)$, (c) Thermal conductivity ($\ln \kappa^* = \text{ANN}(\alpha_{vdw}, \rho^*, 1/T^*)$),(d) Shear viscosity ($\ln \eta^* = \text{ANN}(\alpha_{vdw}, \rho^*, 1/T^*)$, trained with penalty functions (Eqs. (16)).

3.3 $\lambda_r = 30$ and $\lambda_a = 6$

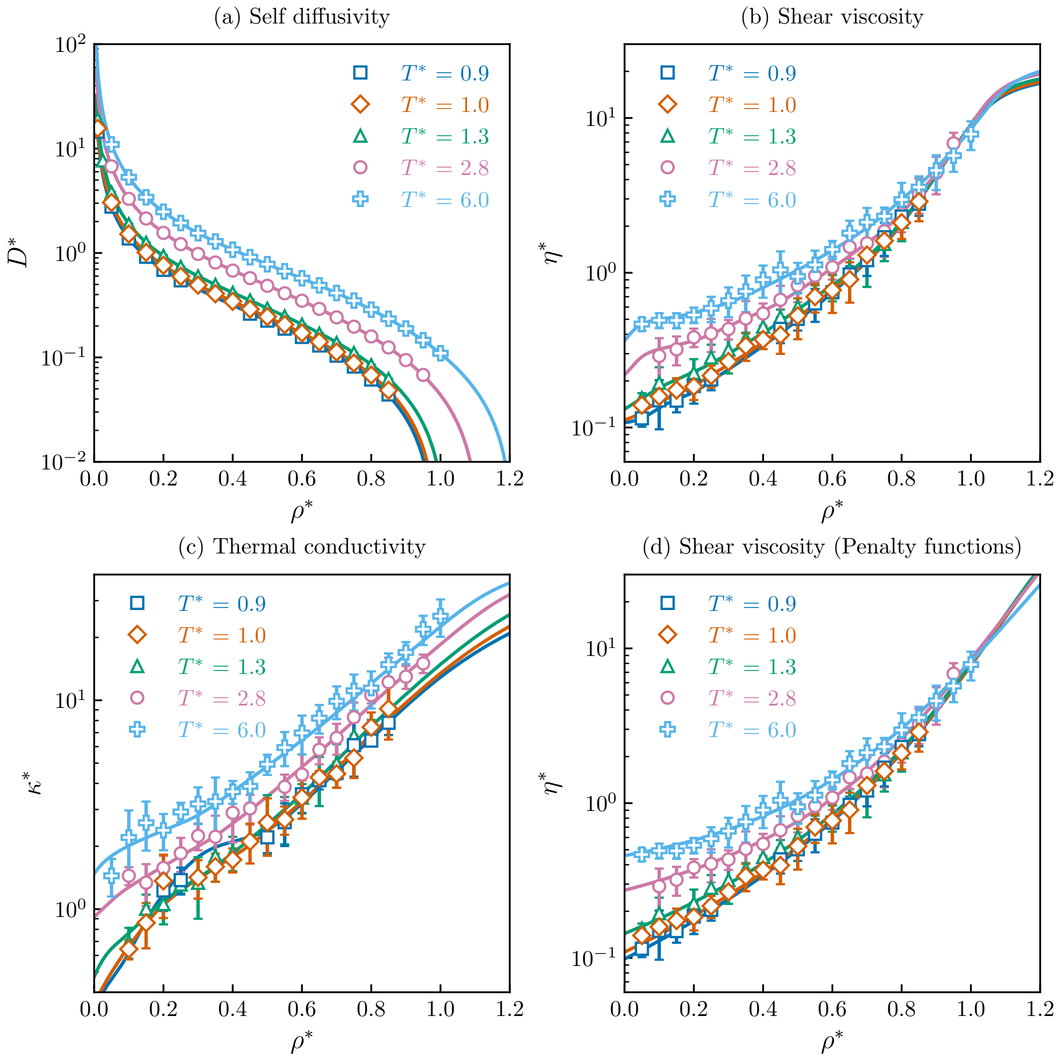

Figure S8: Transport properties isotherms for the Mie fluid ($\lambda_r = 30$ and $\lambda_a = 6$). Molecular dynamics data: Blue squares: $T^* = 0.9$, orange diamonds: $T^* = 1.0$, green triangles: $T^* = 1.3$, pink circles: $T^* = 2.8$ and sky blue plus sign: $T^* = 6.0$. Solid lines: (a) Self diffusivity ($\rho^* D^* = \mathrm{ANN}(\alpha_{vdw}, \rho^*, 1/T^*)$), (b) Shear viscosity ($\ln \eta^* = \mathrm{ANN}(\alpha_{vdw}, \rho^*, 1/T^*)$), (c) Thermal conductivity ($\ln \kappa^* = \mathrm{ANN}(\alpha_{vdw}, \rho^*, 1/T^*)$), (d) Shear viscosity ($\ln \eta^* = \mathrm{ANN}(\alpha_{vdw}, \rho^*, 1/T^*)$, trained with penalty functions (Eqs. (16)).

4 Dilute-gas transport properties of $\lambda_r - 6$ Mie fluids

The transport properties of a dilute-gas can be obtained from kinetic theory.[S2,S3] The first order approximation of the self diffusivity, shear viscosity and thermal conductivity are shown in Eqs. 18.

$$\rho^* D^*_{[1]} = \frac{3}{8}\frac{1}{\Omega^{(1,1)*}}\sqrt{\frac{T^*}{\pi}} \tag{18a}$$

$$\eta^*_{[1]} = \frac{5}{12}\frac{1}{\Omega^{(2,2)*}}\sqrt{\frac{T^*}{\pi}} \tag{18b}$$

$$\kappa^*_{[1]} = \frac{25}{32}\frac{C_V^*}{\Omega^{(2,2)*}}\sqrt{\frac{T^*}{\pi}} \tag{18c}$$

Here, $D^*_{[1]}$, $\eta^*_{[1]}$ and $\kappa^*_{[1]}$ are the first-order approximation for the self diffusivity, shear viscosity, and thermal conductivity, respectively, λ_r is the repulsive exponent of the Mie potential, ρ^* is the dimensionless density, T^* is the reduced temperature and $C_V^* = C_V/R$ is the dimensionless isochoric heat capacity, equal to 1.5 for monoatomic molecules at zero density. Finally, $\Omega^{(l,s)*}$ refers to a collision integral.[S2,S3] Fokin et al.[S4] have calculated and correlated the collision integrals ($\Omega^{(1,1)*}$ and $\Omega^{(2,2)*}$) for $\lambda_r - 6$ Mie fluids. The collision integral $\Omega^{(k,k)*}$ is obtained as follows:

$$\ln \Omega^{(k,k)*} = -\frac{2}{\lambda_r}\ln T^* + \delta^{(k,k)} \ln\left(1 - \frac{2}{3\lambda_r}\right) + \sum_{i=1}^{6} a_i^{(k,k)}(\lambda_r)\left(\frac{1}{T^*}\right)^{(i-1)/2} \tag{S.3}$$

Here, $\delta^{(1,1)} = 0$ and $\delta^{(2,2)} = 1$. The coefficients $a_i^{(k,k)}(\lambda_r)$ are obtained from the following expansion and the parameters $a_{ij}^{(k,k)}$ are found in Table S1.

$$a_i^{(k,k)}(\lambda_r) = a_{i1}^{(k,k)} + \frac{a_{i2}^{(k,k)}}{\lambda_r} + \frac{a_{i3}^{(k,k)}}{\lambda_r^2} + \frac{a_{i4}^{(k,k)}}{\lambda_r^3} \tag{S.4}$$

Table S1: Coefficients for Eq. (S.4) **correlated by Fokin et al.**[S4]**.**

$a^{(k,k)}$	$k=(1,1)$	$k=(2,2)$	$a^{(k,k)}$	$k=(1,1)$	$k=(2,2)$
a_{11}	0.0	0.0	a_{41}	0.485 352	0.697 682
a_{12}	$-0.145\,269\times10^{1}$	$0.113\,086\times10^{1}$	a_{42}	$0.245\,523\times10^{2}$	$0.590\,192\times10^{2}$
a_{13}	$0.294\,682\times10^{2}$	$0.234\,799\times10^{2}$	a_{43}	$-0.336\,782\times10^{3}$	$-0.143\,670\times10^{3}$
a_{14}	$0.242\,508\times10^{1}$	$0.310\,127\times10^{1}$	a_{44}	$0.814\,187\times10^{2}$	$-0.123\,518\times10^{3}$
a_{21}	$0.107\,782\times10^{-1}$	0.0	a_{51}	−0.385 355	−0.564 238
a_{22}	0.587 725	$0.551\,559\times10^{1}$	a_{52}	$-0.206\,868\times10^{2}$	$-0.430\,549\times10^{2}$
a_{23}	$-0.180\,714\times10^{3}$	$-0.137\,023\times10^{3}$	a_{53}	$0.132\,246\times10^{3}$	0.0
a_{24}	$0.595\,694\times10^{2}$	$0.185\,848\times10^{2}$	a_{54}	0.0	$0.137\,282\times10^{3}$
a_{31}	$0.546\,646\times10^{-1}$	$0.325\,909\times10^{-1}$	a_{61}	$0.847\,232\times10^{-1}$	0.126 508
a_{32}	$-0.651\,465\times10^{1}$	$-0.292\,925\times10^{2}$	a_{62}	$0.521\,812\times10^{1}$	$0.104\,273\times10^{2}$
a_{33}	$0.374\,457\times10^{3}$	$0.243\,741\times10^{3}$	a_{63}	$-0.181\,140\times10^{2}$	$0.150\,601\times10^{2}$
a_{34}	$-0.137\,807\times10^{3}$	0.0	a_{64}	$-0.747\,215\times10^{1}$	$-0.408\,911\times10^{2}$

5 Transport properties modeling of selected $\lambda_r - 6$ Mie fluids (residual models)

In the case of $\lambda_r - 6$ Mie fluids, the following models have been proposed. These fulfill exactly the first order approximation of a transport property at zero density, which is obatined from Eqs. (18).

$$\rho^* D^* = \rho^* D^*_{[1]} + [\mathrm{ANN}(\alpha_{vdw}, \rho^*, 1/T^*) - \mathrm{ANN}(\alpha_{vdw}, \rho^* = 0, 1/T^*)] \tag{19a}$$

$$\ln \eta^* = \ln \eta^*_{[1]} + [\mathrm{ANN}(\alpha_{vdw}, \rho^*, 1/T^*) - \mathrm{ANN}(\alpha_{vdw}, \rho^* = 0, 1/T^*)] \tag{19b}$$

$$\ln \kappa^* = \ln \kappa^*_{[1]} + [\mathrm{ANN}(\alpha_{vdw}, \rho^*, 1/T^*) - \mathrm{ANN}(\alpha_{vdw}, \rho^* = 0, 1/T^*)] \tag{19c}$$

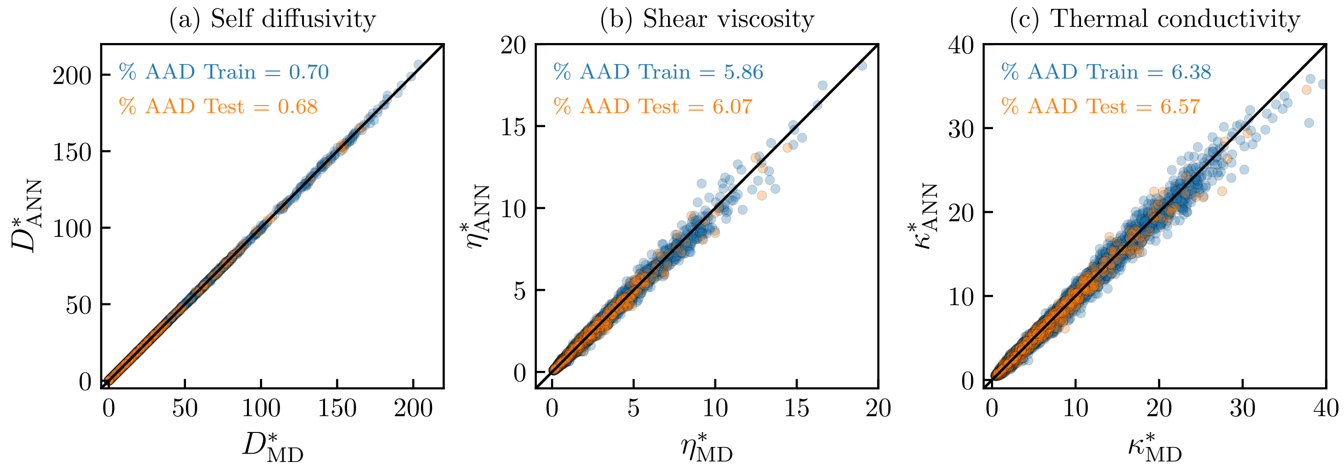

Figure S9: Parity plots for the trained transport properties ANNs models for the Mie fluid with respect to molecular dynamics data. Train data: blue circles, test data: orange circles. (a) Self diffusivity (Eq. (19a)), (b) Shear viscosity (Eq. (19b), trained with penalty functions (Eqs. (16)), (c) Thermal conductivity (Eq. (19c)).

5.1 $\lambda_r = 12$ and $\lambda_a = 6$

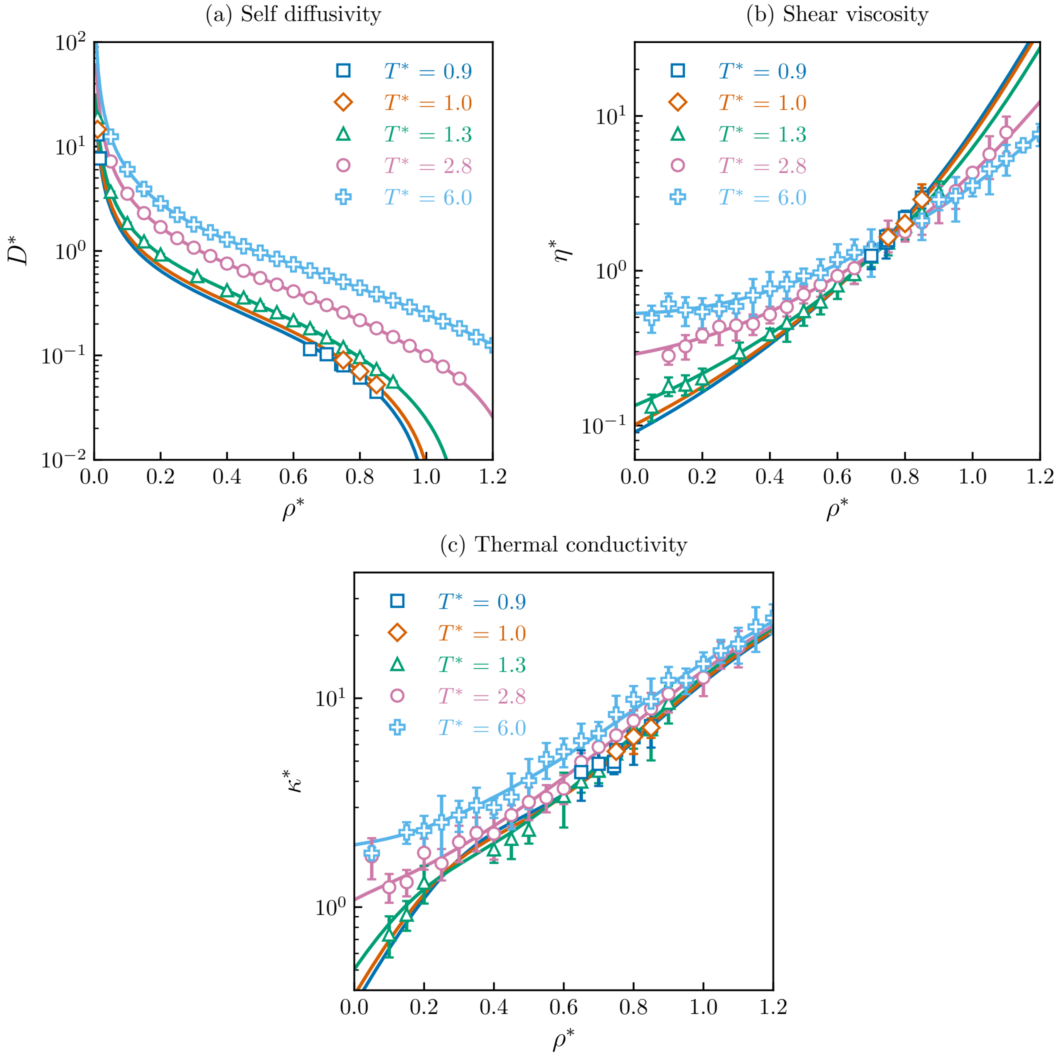

Figure S10: Transport properties isotherms for the Mie fluid ($\lambda_r = 12$ and $\lambda_a = 6$). Molecular dynamics data: Blue squares: $T^* = 0.9$, orange diamonds: $T^* = 1.0$, green triangles: $T^* = 1.3$, pink circles: $T^* = 2.8$ and sky blue plus sign: $T^* = 6.0$. Solid lines: (a) Self diffusivity (Eq. (19a)), (b) Shear viscosity (Eq. (19b), trained with penalty functions (Eqs. (16)), (c) Thermal conductivity (Eq. (19c)).

5.2 $\lambda_r = 18$ and $\lambda_a = 6$

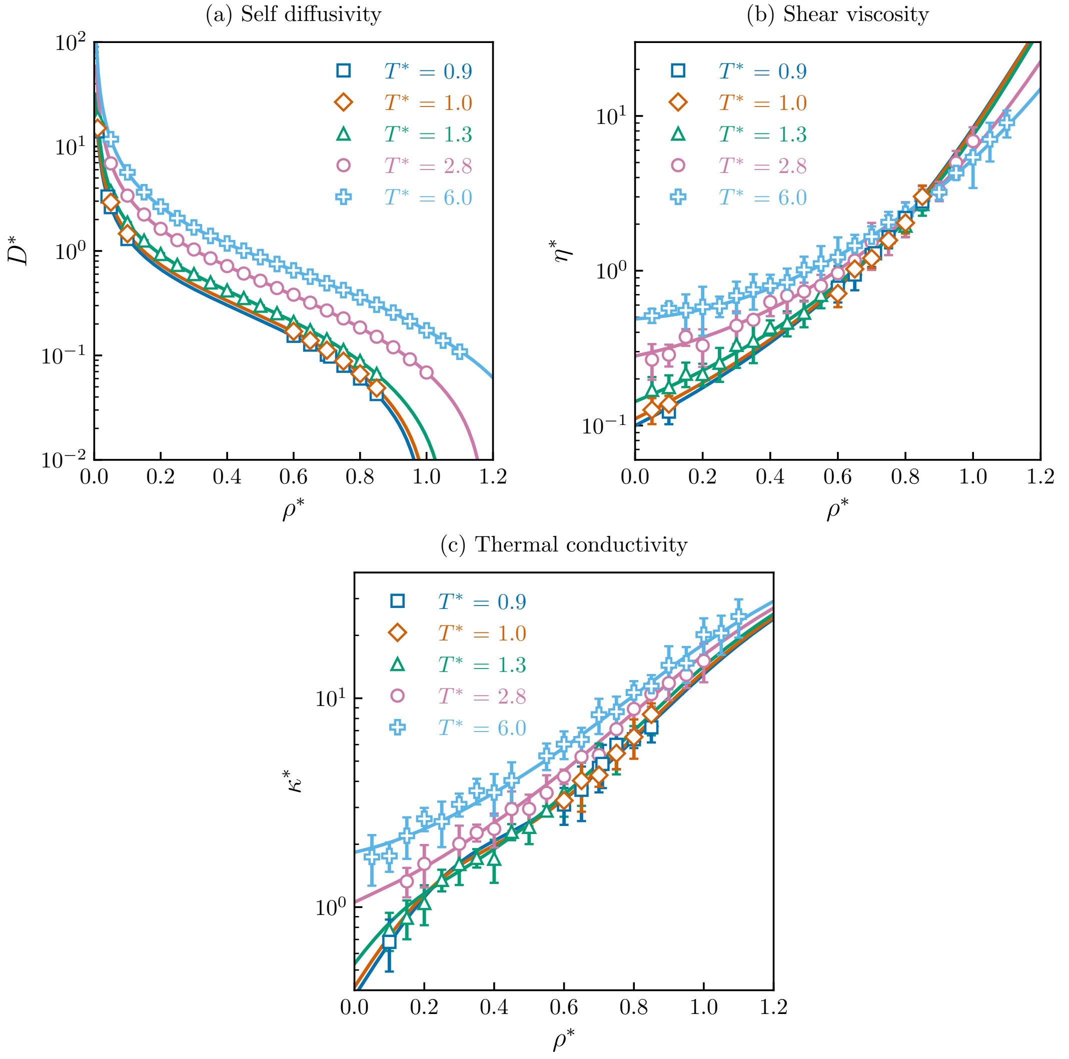

Figure S11: Transport properties isotherms for the Mie fluid ($\lambda_r = 18$ and $\lambda_a = 6$). Molecular dynamics data: Blue squares: $T^* = 0.9$, orange diamonds: $T^* = 1.0$, green triangles: $T^* = 1.3$, pink circles: $T^* = 2.8$ and sky blue plus sign: $T^* = 6.0$. Solid lines: (a) Self diffusivity (Eq. (19a)), (b) Shear viscosity (Eq. (19b), trained with penalty functions (Eqs. (16)), (c) Thermal conductivity (Eq. (19c)).

5.3 $\lambda_r = 24$ and $\lambda_a = 6$

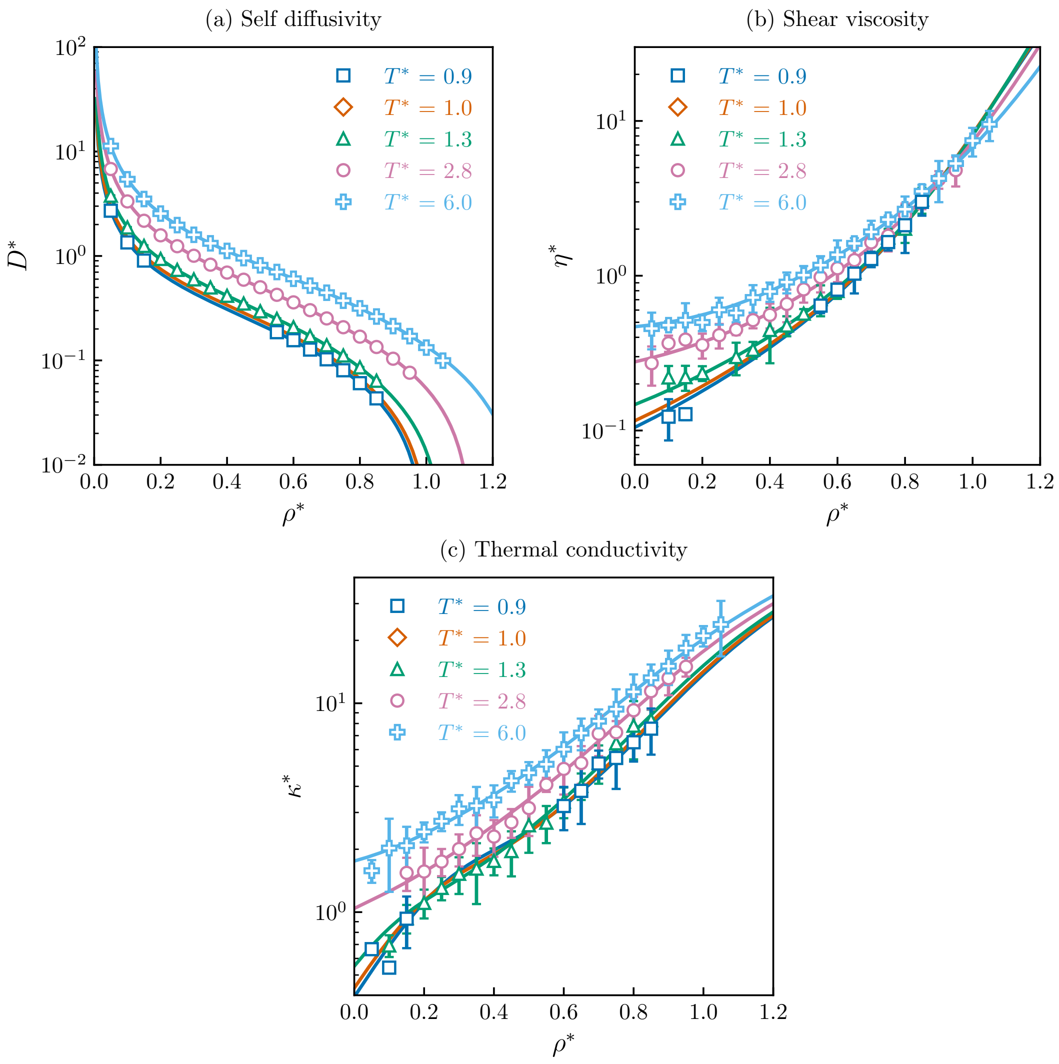

Figure S12: Transport properties isotherms for the Mie fluid ($\lambda_r = 24$ and $\lambda_a = 6$). Molecular dynamics data: Blue squares: $T^* = 0.9$, orange diamonds: $T^* = 1.0$, green triangles: $T^* = 1.3$, pink circles: $T^* = 2.8$ and sky blue plus sign: $T^* = 6.0$. Solid lines: (a) Self diffusivity (Eq. (19a)), (b) Shear viscosity (Eq. (19b), trained with penalty functions (Eqs. (16)), (c) Thermal conductivity (Eq. (19c)).

5.4 $\lambda_r = 30$ and $\lambda_a = 6$

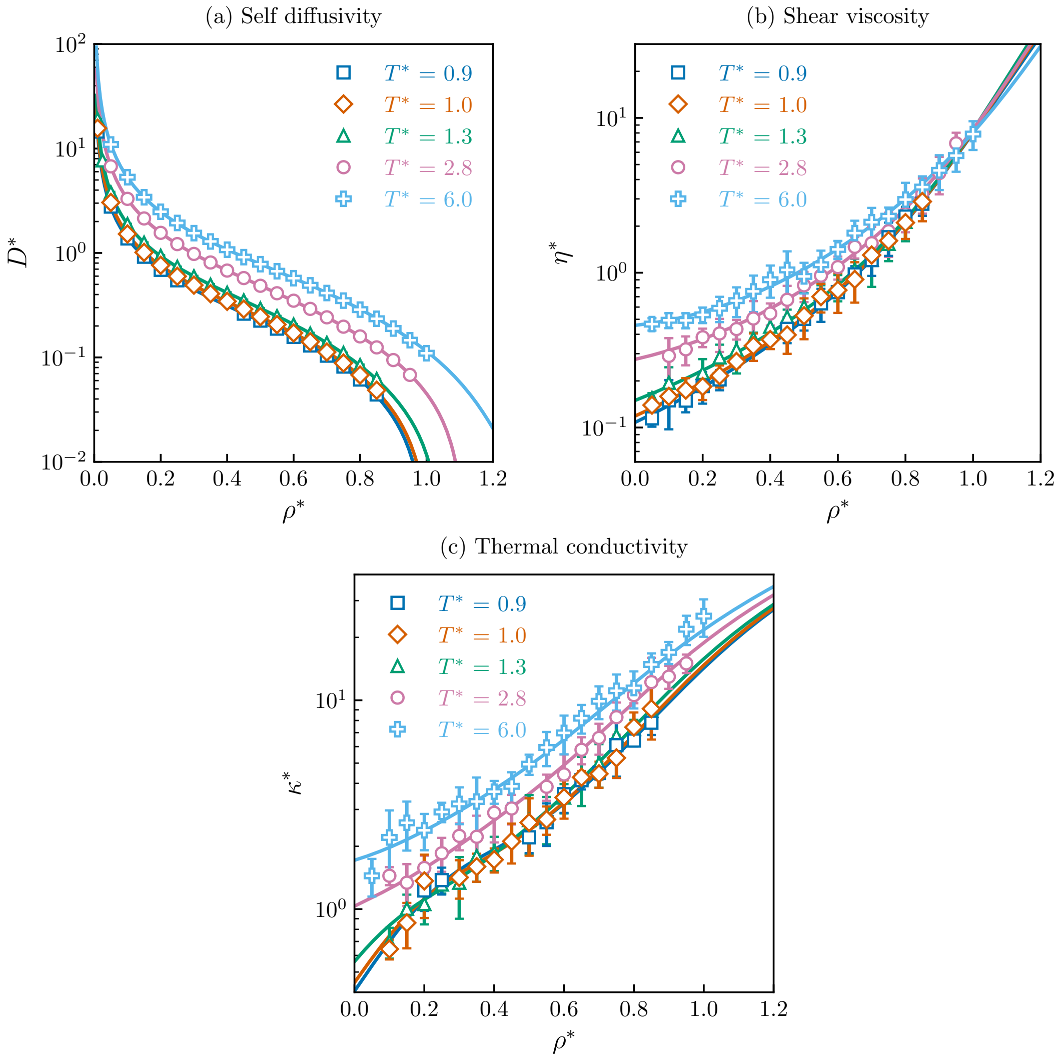

Figure S13: Transport properties isotherms for the Mie fluid ($\lambda_r = 30$ and $\lambda_a = 6$). Molecular dynamics data: Blue squares: $T^* = 0.9$, orange diamonds: $T^* = 1.0$, green triangles: $T^* = 1.3$, pink circles: $T^* = 2.8$ and sky blue plus sign: $T^* = 6.0$. Solid lines: (a) Self diffusivity (Eq. (19a)), (b) Shear viscosity (Eq. (19b), trained with penalty functions (Eqs. (16)), (c) Thermal conductivity (Eq. (19c)).

References

(S1) Akiba, T.; Sano, S.; Yanase, T.; Ohta, T.; Koyama, M. Optuna: A Next-generation Hyperparameter Optimization Framework. *Proceedings of the ACM SIGKDD International Conference on Knowledge Discovery and Data Mining* **2019**, 2623–2631, Submission date: 2019-07-25, arXiv: 1907.10902, Accessed: 2023-10-12.

(S2) Reed, T. M.; Gubbins, K. E. *Applied statistical mechanics: thermodynamic and transport properties of fluids*; McGraw-Hill: New York, 1973.

(S3) Maitland, G. C.; Rigby, M.; Smith, E. B.; Wakeham, W. A. *Intermolecular Forces: Their Origin and Determination*; Clarendon Press: Oxford, 1981.

(S4) Fokin, L.; Popov, V.; Kalashnikov, A. Analytical presentation of the collision integrals for the (m-6) Lennard-Jones potential in the EPIDIF data base. *High Temperature* **1999**, *37*, 49–55.
